# A deep sequencing tool for partitioning clearance rates following antimalarial treatment in polyclonal infections

**DOI:** 10.1093/emph/eov036

**Published:** 2016-01-27

**Authors:** Nicole Mideo, Jeffrey A. Bailey, Nicholas J. Hathaway, Billy Ngasala, David L. Saunders, Chanthap Lon, Oksana Kharabora, Andrew Jamnik, Sujata Balasubramanian, Anders Björkman, Andreas Mårtensson, Steven R. Meshnick, Andrew F. Read, Jonathan J. Juliano

**Affiliations:** ^1^Department of Ecology and Evolutionary Biology, University of Toronto, Toronto, ON, Canada;; ^2^Division of Transfusion Medicine, Department of Medicine, University of Massachusetts, Worcester, MA, USA;; ^3^Program in Bioinformatics and Integrative Biology, University of Massachusetts, Worcester, MA, USA;; ^4^Department of Parasitology, Muhimbili University of Health and Allied Sciences, Dar Es Salaam, Tanzania;; ^5^Division of Immunology and Medicine, USAMC Armed Forces Research Institute of Medical Sciences, Bangkok, Thailand;; ^6^US Army Medical Component, Armed Forces Research Institute of Medical Sciences, Phnom Penh, Cambodia;; ^7^Division of Infectious Diseases, University of North Carolina School of Medicine, Chapel Hill, NC, USA;; ^8^Malaria Research, Department of Microbiology, Tumor and Cell Biology, Karolinska Institutet, Stockholm, Sweden;; ^9^Centre for Clinical Research Sörmland, Uppsala University, Sweden;; ^10^Department of Women’s and Children’s Health, International Maternal and Child Health (IMCH), Uppsala University, Sweden;; ^11^Department of Epidemiology, Gillings School of Global Public Health, University of North Carolina, Chapel Hill, NC, USA;; ^12^Center for Infectious Disease Dynamics, Department of Biology and Entomology, the Pennsylvania State University, University Park, PA, USA and; ^13^Curriculum in Genetics and Molecular Biology, University of North Carolina, Chapel Hill, NC, USA

**Keywords:** malaria, within-host selection, ecology, amplicon sequencing, artemisinin, drug resistance

## Abstract

**Background and objectives:** Current tools struggle to detect drug-resistant malaria parasites when infections contain multiple parasite clones, which is the norm in high transmission settings in Africa. Our aim was to develop and apply an approach for detecting resistance that overcomes the challenges of polyclonal infections without requiring a genetic marker for resistance.

**Methodology:** Clinical samples from patients treated with artemisinin combination therapy were collected from Tanzania and Cambodia. By deeply sequencing a hypervariable locus, we quantified the relative abundance of parasite subpopulations (defined by haplotypes of that locus) within infections and revealed evolutionary dynamics during treatment. Slow clearance is a phenotypic, clinical marker of artemisinin resistance; we analyzed variation in clearance rates within infections by fitting parasite clearance curves to subpopulation data.

**Results:** In Tanzania, we found substantial variation in clearance rates within individual patients. Some parasite subpopulations cleared as slowly as resistant parasites observed in Cambodia. We evaluated possible explanations for these data, including resistance to drugs. Assuming slow clearance was a stable phenotype of subpopulations, simulations predicted that modest increases in their frequency could substantially increase time to cure.

**Conclusions and implications:** By characterizing parasite subpopulations within patients, our method can detect rare, slow clearing parasites *in vivo* whose phenotypic effects would otherwise be masked. Since our approach can be applied to polyclonal infections even when the genetics underlying resistance are unknown, it could aid in monitoring the emergence of artemisinin resistance. Our application to Tanzanian samples uncovers rare subpopulations with worrying phenotypes for closer examination.

## Abstract (precis)

Our approach for detecting drug resistance identifies rare resistant parasites in polyclonal infections through their phenotypic signature. By translating genetic data to clearance phenotypes of parasite subpopulations in malaria infections, we found rare slow clearing parasites in Tanzania where resistance to front-line drugs is not thought to be a problem.

## INTRODUCTION

The emergence of drug-resistant malaria parasites is a major hurdle in the control of *Plasmodium falciparum*, which has evolved resistance to nearly every antimalarial drug in use [1]. A major concern is the emergence in South East Asia of resistance to the current front-line artemisinin derivatives [2–6], which could spark a global health crisis. To maximize the continued efficacy of these drugs, early detection of resistance, good surveillance and containment are required.

Drug resistance in *P. falciparum* may be monitored using three tools: (i) therapeutic efficacy tests, (ii) *in vitro* susceptibility tests and (iii) molecular markers that are linked to the determinants of resistance. Each of these approaches has advantages and limitations [7]. For artemisinin resistance, significant advances in these areas have been made over the last several years, with a strong molecular correlate for resistance identified in Asia [8] and *in vitro* sensitivity assays developed [9]. The polymorphisms in a gene (*kelch13*) associated with artemisinin resistance in Asia have been observed in Africa [10, 11], though their associated phenotypes in African parasites have not yet been identified. Molecular assays are complicated by the fact that the resistance phenotype associated with some K13 mutations depends on the genetic background in which they occur, suggesting a multi-gene trait [12], and there is evidence that compensatory mutations may be important for the selection and spread of these mutations [13]. Finally, there may be alternate pathways to artemisinin resistance, as was observed for resistance to chloroquine [14], so the genetic signature of any artemisinin-resistant parasites that emerge in Africa may be different than what is emerging in Southeast Asia (as suggested by recent data [15–17]). Thus, phenotypic data—either *in vivo* or *in vitro*—is still required for detecting resistance to artemisinin [18].

**Figure 1.**
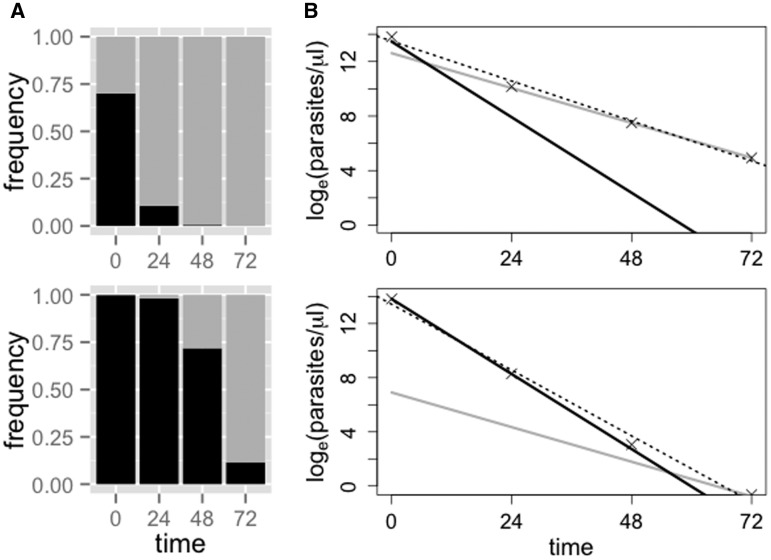
**Clearance curves and determination of ‘clones’ by deep sequencing.** Predicted relative abundances (**A**) and clearance curves (**B**) for hypothetical infections composed of sensitive (black, 3-h half-life) and resistant (gray, 6.5-h half life) parasite clones after drug treatment. The initial frequency of the resistant clone is 25% in the top row and 1% in the bottom row. In (B), the dotted line shows the standard parasite clearance curve, as fitted to the total parasite density. The half-life estimates from these curves, for the whole infection, are ∼5.5 h (top) and ∼4 h (bottom). When the resistant clone is rare it exerts little effect on the overall estimate of parasite half-life

The current clinical tool for phenotyping parasites and monitoring therapeutic efficacy of artemisinin is the parasite clearance curve, which tracks the decline in total parasite density over the course of treatment [19, 20]. Infections that have slower clearance times (longer half-lives) are regarded as harboring more resistant parasites [2–4]. However, parasite clearance curves are less informative in regions with relatively high complexity of infection (i.e. many parasite clones or strains within a host) since the parasite clearance curve is a whole-infection level measure that represents a weighted average of the clearance phenotypes of different parasites within that sample [7]. Recent work in Southeast Asia, where artemisinin resistance is emerging, has shown individual infections harboring parasites with different and stable clearance phenotypes [21]. In Africa, the majority of falciparum malaria infections are polyclonal [22] meaning that resistant parasites are likely to share their host with sensitive parasites [23–26], particularly during the early spread of drug resistance. When resistance is rare, the effects of slow clearing clones on infection-level estimates of resistance are likely to be obscured by the sheer number of faster-clearing, sensitive parasites in an infection (Fig. 1) [7]. Understanding the consequences of drug treatment on the within-host evolutionary dynamics of malaria infections in Africa is critical for understanding the risks of artemisinin resistance emerging.

To overcome the challenges imposed by polyclonal infections, we previously proposed an approach that capitalizes on second generation sequencing to reveal phenotypic signatures of resistance by partitioning an infection into subpopulations of parasites [7]. Our approach characterizes and quantifies the relative abundance of subpopulations by sequencing a highly polymorphic region to define haplotypes and their frequencies over the course of drug treatment [7, 11, 22, 25, 27]. A subpopulation may represent one or multiple clones (genetically identical parasites that can only be truly defined by extensive whole-genome sequencing of individual parasites) and while these subpopulations may still be composed of phenotypically variable parasites, they offer considerably higher resolution than whole-infection measures of clearance phenotypes. The number of haplotypes we detect [22] is on par with clone estimates from whole-genome sequencing [28] and currently exceeds other genotyping methods in similar parasite populations [25]. Importantly, identifying drug-resistant haplotypes using this approach does not require genetic linkage (or physical proximity) of the chosen marker to the genetic determinant of resistance. Instead, our approach makes it possible to detect whether parasites with the resistance phenotype, i.e. slow clearance, are present in infections with sensitive parasites because subpopulations composed of predominately resistant clones will increase in relative abundance as drug treatment is applied (Fig. 1). It also allows for *post hoc* genetic work, by identifying slower clearing subpopulations that demand further study.

We now apply and refine our approach by investigating the clearance phenotypes of parasite subpopulations longitudinally through the initial days of drug treatment from two populations: (i) Cambodian participants treated with dihydroartemisinin-piperaquine (DHA-Pip) with parasite clearance times of 72 h or greater by microscopy and (ii) Tanzanian participants treated with artemether-lumefantrine (AL) with parasite clearance times less than 72 h by microscopy but polymerase chain reaction (PCR)-detectable residual parasitemia up to 72 h. We deep sequenced the region of the *P. falciparum* circumsporozoite protein gene (*pfcsp*) encoding the polymorphic C-terminal region, generating haplotypes that defined the subpopulations, which were quantified and tracked during the course of infection. Though this is an immunogenic antigen, this gene is not expressed by parasites in the erythrocytic stage that we are studying, which helps minimize the impact anti-csp immunity may play on clearance rates. We show that within complex falciparum infections in Tanzania, *pfcsp* subpopulations demonstrate significant variation in their response to artemisinin-combination therapies (ACTs) and that some subpopulations clear at rates as slow as those of artemisinin-resistant parasites in Cambodia. We also find that the slowest clearing subpopulations sampled in Tanzania do not contain polymorphisms in the gene associated with artemisinin resistance in Southeast Asia [8].

## METHODOLOGY

### Ethics statement

The trials from which clinical specimens were used were reviewed by ethics boards of the National Institute for Medical Research, Dar es Salaam, Tanzania; Regional Ethics Committee, Stockholm, Sweden; Walter Reed Army Institute of Research; National Ethics Committee for Health Research in Cambodia and University of North Carolina [29, 30].

### Study participants

Cambodian samples were taken from an *in vivo* efficacy study of DHA-Pip [29]. This trial was registered with the ClinicalTrials.gov identifier NCT01280162. Samples from all participants with clearance times of 72 h or greater by microscopy (seven patients) were used. Tanzanian samples were selected from ones obtained from 50 children with uncomplicated falciparum malaria from Fukayosi, Bagamoyo District, Tanzania, that were enrolled in a trial of AL in 2006 [30]. Samples from all participants who remained PCR positive at 72 h (seven patients) were used. In addition, samples from patients who cleared (i.e. were PCR negative) after 48 h (seven patients) and a subset of samples from patients who cleared after 24 h (5 patients) were included.

### Sequencing of *pfcsp*

**Figure 2.**
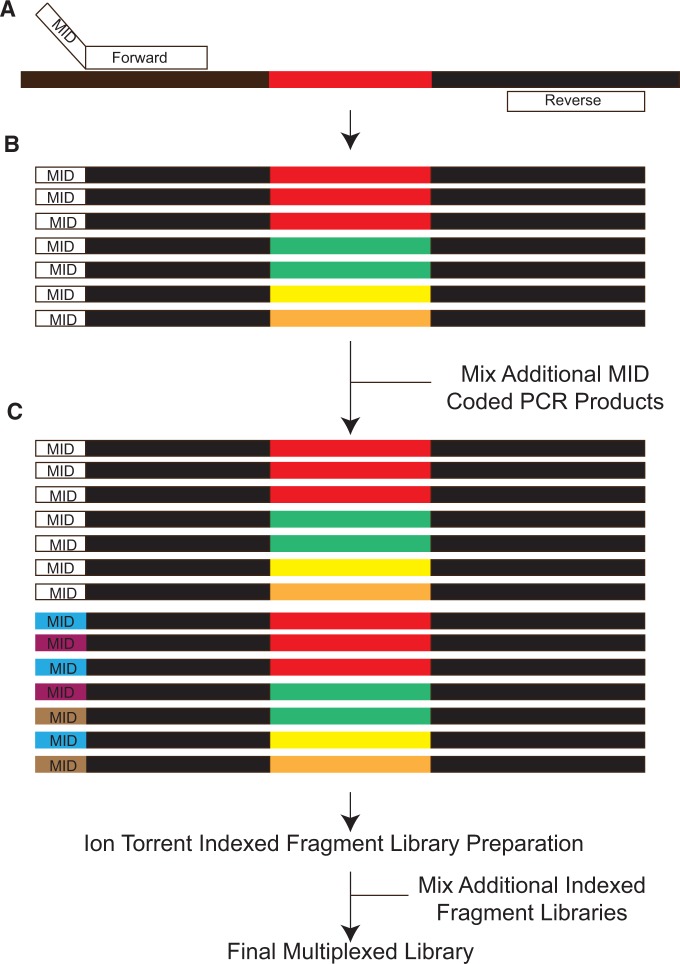
**Schematic of library preparation for highly multiplexed amplicon deep sequencing.** (**A**) Samples are amplified using primers targeting conserved sequences (black fragments) surrounding variable regions (colored fragments). The forward primer contains a barcode (MID) specific to the sample and replicate. (**B**) After amplification, each PCR reaction contains amplicons representing the haplotypes within that sample labeled with the specific MID. (**C**) Multiple samples are then mixed forming a final library containing multiple MIDs (white, blue, purple and brown) for preparation for sequencing. This mixture is library prepped with a specific index placed on the library during the process. Multiple libraries with different indexes can then be pooled and sequenced at the same time allowing for highly multiplexed sequencing

Genomic DNA was extracted from filter paper blood spots using an Invitrogen Pure-link 96-well Genomic DNA extraction kit (Invitrogen, Carlsbad, CA). The C-terminal region of the gene *pfcsp* was amplified using primers previously described [22], with a minor modification. The samples were amplified using a sample-specific forward primer indexed with a barcode, based on published molecular identifier (MID) sequences from Roche, and a universal non-indexed reverse primer. All samples were amplified and sequenced in duplicate. The PCR was carried out using 300 nm of each primer using Q5 HiFidelity polymerase (New England Biolabs, Ipswich, MA) under the conditions previously published [22]. Each sample was amplified in duplicate, and equimolar amounts of each barcoded PCR product (after 35 cycles) were mixed. These libraries were prepared for sequencing on an Ion Torrent PGM at the UNC Microbiome Core Facility using the Ion Plus Fragment kit (Life Technologies, Foster City, CA) and IonExpress Index Barcodes. Equimolar amounts of each library were pooled and sequenced using the 400-bp sequencing kit. By incorporating two barcodes, one in the PCR and one in the library preparation, we can highly multiplex the sequencing of amplicons (Fig. 2). Sequence reads were demultiplexed and clustered using an in-house script, SeekDeep, as previously described [31] (http://baileylab.umassmed.edu/SeekDeep). Haplotypes were kept if they occurred in both duplicate sequencing reactions and were above an averaged frequency cutoff of 0.5% (i.e. if the average frequency across duplicate runs was above this cutoff). This was the default cutoff; however, we defined parasite density-specific cutoffs to account for the fact that sequencing is more error prone when parasite density is lower by generating a dilution series of control mixtures (described below). For each sample (patient and time point), we defined a unique average frequency cutoff, dependent on the parasite density in that sample.

### Quantification of parasitemias

The quantification of parasite DNA in all extracted samples was done using a Biorad QX200 digital droplet PCR system (BioRad, Hercules, CA) allowing for absolute quantification of parasitemias in the extracted DNA [32]. Primers and probes for PgMet were adapted from previous reports [33]. The assay was completed using primers at a concentration of 300 nM and probe at a concentration of 200 nM using the ddPCR Supermix for probes.

### Control mixtures and sequencing dilution series

Stocks of malaria DNA from MR4 were quantified using a previously described real-time PCR assay [34]. A mixture was created using stocks with three different *pfcsp* genotypes in the region amplified for deep sequencing. This control DNA was mixed with human DNA to reach a final concentration of 1 ng/µl. The sample was then diluted 1:2 using human DNA to maintain the final total concentration of 1 ng/µl while reducing the amount of malaria DNA in the sample. Parasite density is expressed as genome equivalents (GE) per microliter, calculated using the approximate genome size of 23 Mb and an average base pair weight of 650 Daltons. This provided control samples ranging from 1536 to 3 GE/µl. Each sample in the dilution series was then amplified using 6 MID labeled primers. PCR fragments were library prepped as previously described.

To confirm the contents of the mixture, the PCR product from one of the mixtures was cloned into a pCR4-TOPO plasmid using the TOPO TA Cloning Kit for Sequencing (Invitrogen). Colonies were picked and plasmids harvested using the Zyppy-96 Plasmid Miniprep (Zymo Research) and cloned fragments were Sanger Sequenced at Eton Biosciences (RTP, NC). Parasite density-specific frequency cutoffs were determined by plotting the parasite density of control mixtures versus the frequency of false haplotypes observed in those mixtures. We fitted a non-linear curve to this data and used the fitted curve to predict the frequency below which calling haplotypes would be suspect, for a given input density. See Supplementary Material for fitted curve and further details.

### Sequencing of the K13-propeller domain

We used a hemi-nested protocol to amplify nucleotides 1279–2030 of *PF3D7_1343700*, corresponding to the K13-propeller domain as previously described [11]. Products were electrophoresed on 2% agarose gels to confirm products. PCR products were cleaned with the Invitrogen PureLink kit (Life Technologies, Carlsbad, CA), and these were bi-directionally sequenced using ABI BigDye Terminator chemistry at Eton Bioscience (San Diego, CA). Reads were aligned to reference *PF3d7_1343700* (www.plasmodb.org; 11 January 2016, date last accessed) and scored for polymorphisms.

### Genetic variation in clearance curves

Clearance curves for individual parasite subpopulations (as defined by haplotypes) were produced by multiplying total parasitemia (quantified by PCR) by the relative abundance of those subpopulations (quantified by amplicon sequencing). This generated two density estimates for each subpopulation at each time point since there were technical replicates at the PCR and sequencing stages, with DNA extracted from a single blood spot. For each patient that was sampled at a minimum of two time points, we fitted two linear models to the subpopulation-specific decline in log_e_ parasitemia over time (using the lm function in R, version 3.0.2; http://www.R-project.org). Model 1 included subpopulation as a fixed factor, as well as an interaction between subpopulation and time, allowing for unique slopes and intercepts for each subpopulation. Model 2 included subpopulation as a fixed factor but no interaction term. By allowing for unique intercepts, but a single clearance slope, Model 2 assumes that all subpopulations are cleared from the infection at the same rate, i.e. are equally sensitive to drug treatment. Included in these analyses were only those subpopulations that occurred at time 0 and were sufficiently abundant to be captured by sequencing in at least one other time point (there are no fitted lines for some of the subpopulations). We did this to avoid making arbitrary assumptions about the initial frequency of subpopulations that emerged later. Additionally, we suggest that this will provide a conservative estimate of resistance within a host, as any initially undetectable subpopulation that emerges at later time points likely has an even slower clearance rate than those we do estimate.

For each patient, we compared the change in deviance between Model 1 and Model 2 to F distributions. Degrees of freedom correspond to the difference in the number of terms in the model relative to the residual degrees of freedom. Significant changes in deviance indicate that the more complicated model (Model 1) provides a better fit to the data and therefore demonstrates significant variation in clearance rates. We found significant temporal autocorrelation within two of the individual datasets (T01, *P* = 0.025; T47, *P* = 0.0002), so we reran these analyses using Generalized Least Squares models, incorporating a corAR1 autocorrelation structure in the fitted models [35, 36]. Statistical analyses were performed using R version 3.0.2. Only results from patients who were PCR positive at 72 h post-drug treatment are included in the main text. Similar analyses and results are presented in the Supplementary Material for patients who were PCR negative at or before 72 h post-drug treatment.

### Estimating biological significance of variation in clearance curves

To determine whether the level of variation in clearance rates observed is biologically important, we simulated infections with two clearance phenotypes to see how changing the frequency of slow clearing parasites would affect time to clearance. For each of the six patients presented in the main text for whom we found significant variation in clearance rates, we simulated infections with the same initial parasitemia as was measured for that patient at time 0 in the clinic. These parasites were divided into two phenotypes: one clearing at a rate estimated directly from fitting a linear model to the log_e_ parasitemia measured by PCR (i.e. the weighted average clearance rate of the whole infection, as would be estimated by the Parasite Clearance Curve Estimator, www.wwarn.org/pce/) [19] and one clearing at a rate given by the shallowest slope estimated for any subpopulation in that infection (i.e. the slowest clearing subpopulation). Assuming the same log-linear decline due to drugs, the log_e_ density of parasites with phenotype *i* (slow clearing or average) at time *t* in hours, *P_i_*_,_*_t_*, is given by

$$P_{i,t}=\text{log}\left(f_{i}D_{0}\right)+m_{i}t,$$

where *f_i_* is the initial frequency of parasites with phenotype *i*, *D*_0_ is the initial (unlogged) total parasite density and *m_i_* is the clearance rate of phenotype *i*. Total parasite density at time *t*, *D_t_*, is given by

$$D_{t}=f_{\text{S}}D_{0}e^{m_{\text{S}}t}+\left(1-f_{\text{S}}\right)D_{0}e^{m_{\text{A}}t},$$

where the *S* and *A* subscripts indicate either a slow clearing or average phenotype, respectively. For a given frequency of the slow clearing phenotype, *f_S_*, we simulated total parasite density over many hours (max. 3000) and determined the time at which the infection was reduced to a density of 1 parasite genome per microliter of sample (the threshold of detection). We then compared this ‘time to clearance by PCR’ with the predicted time to clearance given *f_S_* = 0 (in other words, the predicted time to clearance of the actual infection). The difference in these times to clearance is reported.

## RESULTS

### Control mixtures and determination of parasite density-specific frequency cutoffs

**Figure 3.**
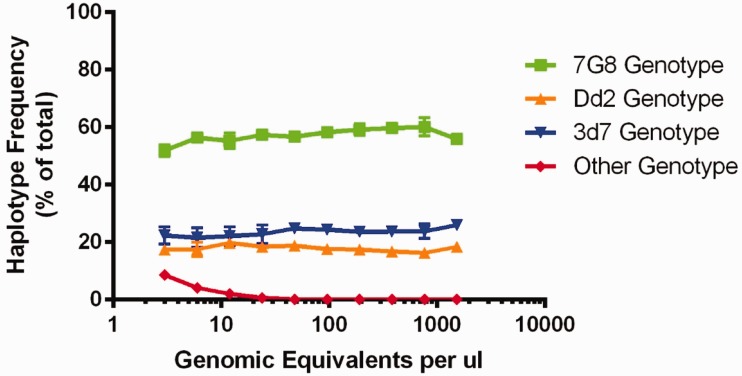
**Deep sequencing of control mixtures.** The detected haplotype frequencies of the dilution series are shown, with parasitemias ranging from three genomic equivalents per microliter (GE/µl) to 1536 GE/µl. Each point represents the mean (dot) and 1 SD (error bars) of triplicate experiments. Each experiment involves two PCR replicates used to call haplotypes with a fixed minimum cutoff frequency of 0.5% using SeekDeep. Overall, there is little variation in the frequency estimates within a concentration and between concentrations. Beginning at 24 GE/µl, false-positive haplotypes begin to be detected. Plotted here is the sum total frequency of false haplotypes (red line). Individual samples could contain between 1 and 4 false haplotypes, which individually never exceeded 6% within a single experiment

Based on the replicate real-time PCR results, the control mixture contained 39.13 ng ± 9.18 ng of 7G8 genotype, 30.56 ng ± 2.45 ng of 3D7 genotype and 25.13 ng ± 4.23 ng of Dd2 genotype prior to dilution. Cloning (*n* = 22) and sequencing of the PCR product confirmed that 12/22 (55%), 6/22 (27%) and 4/22 (18%) of the PCR product was 7G8, 3D7 and Dd2, respectively. The Ion Torrent sequencing results of the dilution series are shown in Fig. 3.

Total parasite density and read counts for all patients at all time points are given in Supplementary Table S1. Supplementary Table S2 lists the haplotype frequency cutoff used for each of those samples. At a minimum, we required that a haplotype accounted for at least 0.5% of the reads in a given sample, a cutoff that has been used in previous studies [37]. Nine out of 26 samples required higher frequency cutoffs than this standard.

### Determination of haplotypes in longitudinal samples

Initial sequencing returned 2 514 710 reads corresponding to the C-terminus of *pfcsp*. After filtering, 1 975 650 (79%) reads were used in our final haplotype construction. On average, sampled time points had a read depth of 9685 (range: 203–45 532). After clustering, we determined the total number of unique *pfcsp* haplotypes across our patient populations with 10 for Cambodian (7 patients) and 41 for Tanzanian (19 patients) samples (Accession Numbers: KR606647-KR606687). All 10 Cambodian haplotypes were found within the more diverse Tanzanian population. The majority of the diversity across haplotypes is contained within the T-cell epitope regions of the gene and has been observed previously in other populations [22, 38]. Across all samples, single-nucleotide polymorphisms were observed at ∼6% of non-epitope sites and 31% of epitope sites. Looking at the subset of samples from given time points, we find similar frequencies: at 0 h post-treatment, 5% non-epitope and 31% of epitope sites are variable; at 72 h, the frequencies are 1% and 26%, respectively. Overall, we observed very few potentially spurious haplotypes due to accumulation of random errors across the sequence, and we found roughly equal numbers at early and late time points. Such haplotypes would appear as an enrichment of novel haplotypes that occur in a single sample, given that the majority of the base positions are invariant. We observed six single occurrence haplotypes in early time points (−2, 0 and 24 h) and five single occurrence haplotypes in late time points (48 and 72 h). There was no trend of increasing numbers with later time points and, in fact, we did not observe any single occurrence haplotypes at 72 h.

**Figure 4.**
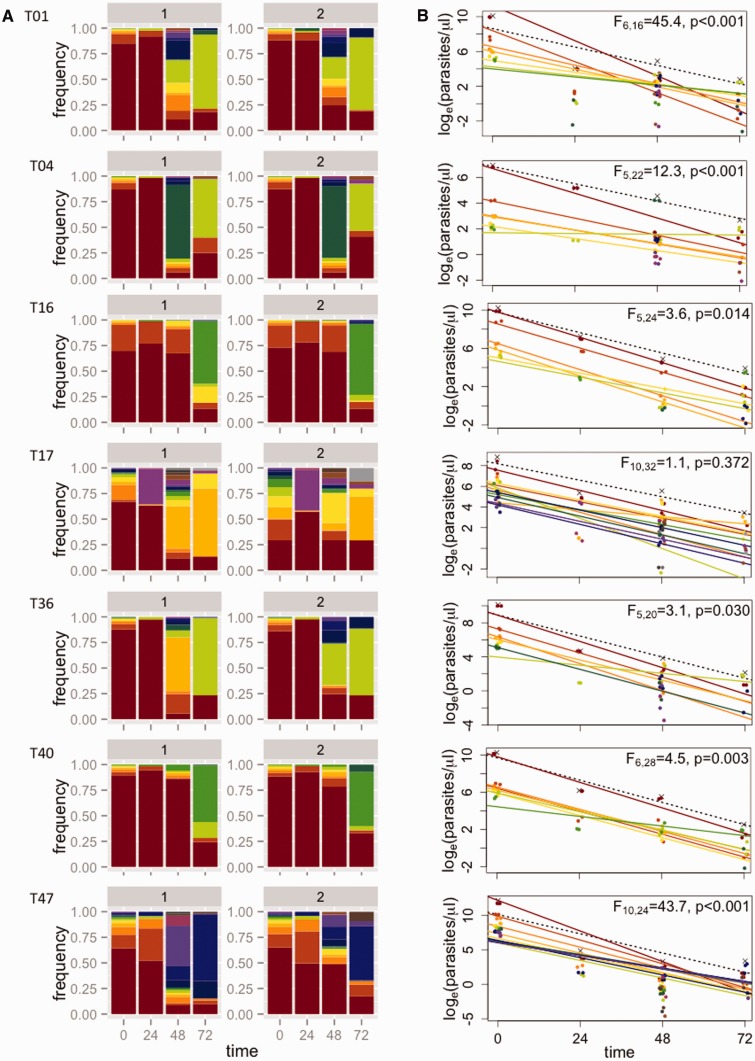
**Estimating subpopulation clearance curves from infections in Tanzania.** (**A**) Relative abundance of different parasite subpopulations (defined by haplotypes) within patients. For each patient (row), there are two bar graphs representing technical replicates, labeled 1 and 2. Within each patient, individual subpopulations have specific colors and are ranked by initial frequency. (The same color may thus represent different haplotypes in different patients.) (**B**) Densities of individual subpopulations (points) are calculated as the total density by quantitative PCR (crosses) multiplied by their relative abundance. Clearance curves are plotted for the total parasite density (dashed lines) and individual subpopulations (colored lines). Statistics reported represent model comparisons; *P* values < 0.05 indicate that a more complicated model that includes different slopes for each subpopulation within a patient explains significantly more variation than a model with a single slope. Similar plots for 12 additional patients for whom parasites could not be detected at 72 h by PCR can be found in the Supplementary Figs S2 and S3

**Figure 5.**
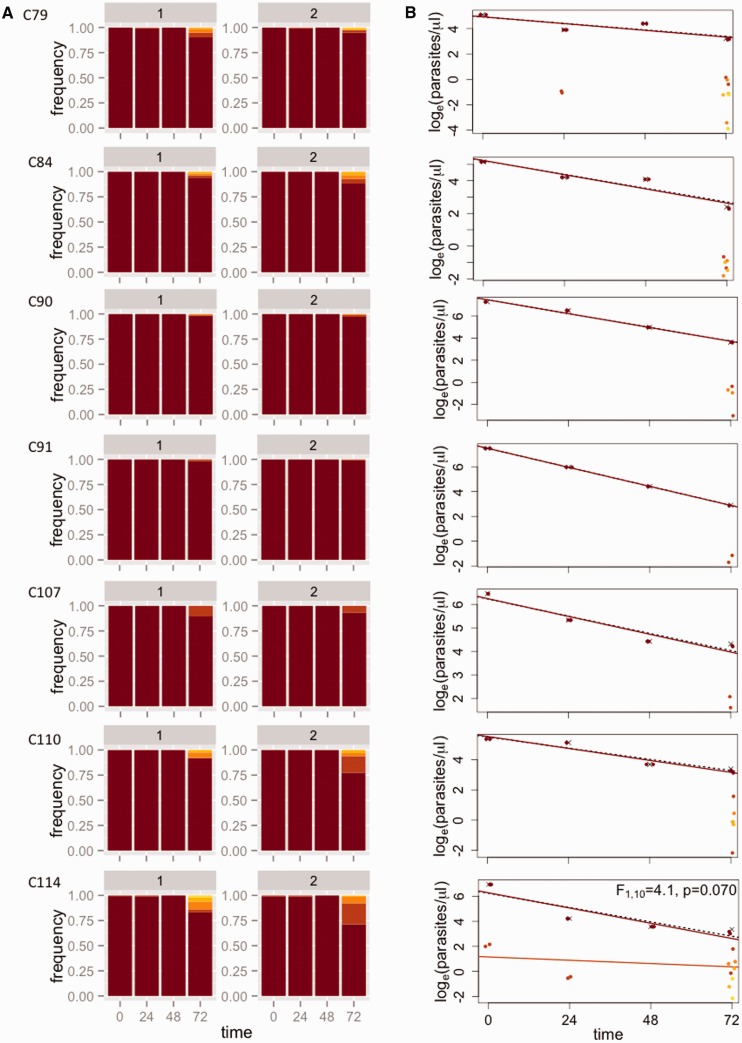
**Estimating subpopulation clearance curves from infections in Cambodia.** (**A**) Relative abundance of different parasite subpopulations (defined by haplotypes) within patients. (**B**) Densities of individual subpopulations (points) are calculated as the total density by PCR (crosses) multiplied by their relative abundance. Clearance curves are plotted for the total parasite density (dashed lines) and individual subpopulations (colored lines)

The frequencies of all subpopulations (defined by the haplotypes) for each patient are given in Supplementary Table S3. Figure 4a shows how the relative abundance of different subpopulations changes over the course of drug treatment for seven Tanzanian patients who were PCR positive 72 h after treatment was initiated. Similar plots for 12 additional patients for whom parasites could not be detected at 72 h by PCR can be found in the Supplementary Material (Supplementary Figs. S2 and S3). Note that for all Tanzanian patients, parasites were undetectable by microscopy 72 h after the start of treatment. In contrast, all seven Cambodian patients were microscopy positive 72 h after the start of treatment; changes in the relative abundance of subpopulations in these infections are shown in Fig. 5a.

**Figure 6.**
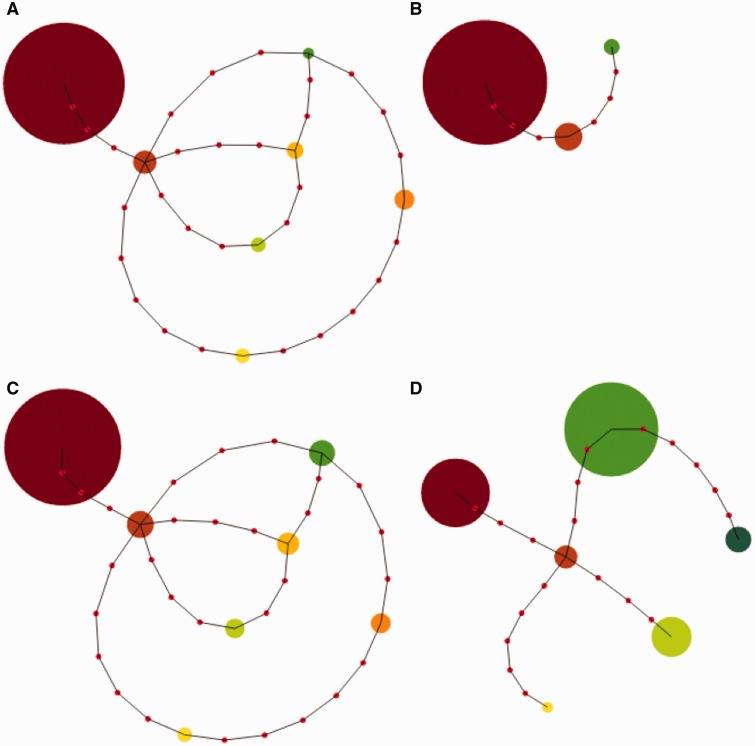
**Genetic relationships between parasite haplotypes in one Tanzanian patient.** Pseudo minimum spanning tree figure showing the number of nucleotide differences (red dots) between haplotypes (circles) with the minimum number of mismatches and their ties shown to include all haplotypes in the graph for patient T40. Haplotypes are colored as in Fig. 3 (second row from the bottom) and the size of the circle corresponds to the relative abundance of that haplotype at (**A**) 0 h, (**B**) 24 h, (**C**) 48 h and (**D**) 72 h after the start of drug treatment. Across time points, the total area of the circles is constant

Figure 6 shows the genetic relationships between haplotypes observed in one Tanzanian patient, as well as their relative abundance over time. Each haplotype found within an individual is highly genetically distant from the other haplotypes within the same individual, suggesting that these represent true differences. In the case of patient T40, the haplotype that was initially most abundant and cleared quickly (dark red) differs by eight nucleotides from the haplotype that was initially rare and cleared most slowly (green). Note that while total parasite density in the infection was decreasing, the total size of the circles in these plots remains constant over time. (The green haplotype is clearing from the infection but increases in relative abundance because it clears at a slower rate.)

### K13-propeller domain analysis

Sequencing of the K13-propeller domain of *PF3D7_1343700* showed the presence of the C580Y mutation associated with artemisinin resistance in all seven Cambodian isolates. The time 0 and 72-h samples from Tanzania were wild type at all positions across the amplified region, with none of the polymorphisms described in Cambodia [8], none previously in the MalariaGen beta release of the *P. falciparum* Community Project (http://www.malariagen.net/apps/pf/2.0/#start) and none previously described in Africa [10, 11].

### Clearance curves of subpopulations

For individual patients, we combined density data over time with the frequency data to plot subpopulation-level clearance curves (Fig. 4b) and compared the fit of two linear models (with and without different slopes for each subpopulation) to these data to test for variation in clearance rates. In all but one patient in Tanzania with residual parasitemia 72 h post-drug treatment, we found evidence of significant variation in clearance rates between subpopulations, i.e. the model including multiple slopes provided a significantly better fit to the data (Fig. 4b). Identical inferences are drawn if models are compared using the Akaike Information Criterion (AIC) instead (Supplementary Table S4). The best-fit linear models provided good fits to the data (mean *R*^2 ^= 0.822 ± 0.039 SE). Similar analyses for 12 patients who were PCR negative 72 h post-drug treatment revealed significant variability in clearance rates in eight of those infections as well (see Supplementary Material and Figs S2 and S3; mean *R*^2^ = 0.879 ± 0.031 SE for all datasets from Tanzania). That we found significant variation in clearance rates despite no PCR-detectable parasites in these infections 72 h after treatment may be due to lower average parasite densities at the start of treatment (the mean parasite density at the start of treatment ± one standard error of the mean for the patients who had no detectable parasites at 72 h was 10 345 ± 3513 and for patients with detectable parasites at 72 h was 42 689 ± 24 073). There was no evidence of variation in clearance rates in the patients from Cambodia. Indeed, most patients harbored a single dominant subpopulation for which a linear curve could be fit to density data (Fig. 5b).

### Biological significance of variation in clearance rates

**Figure 7.**
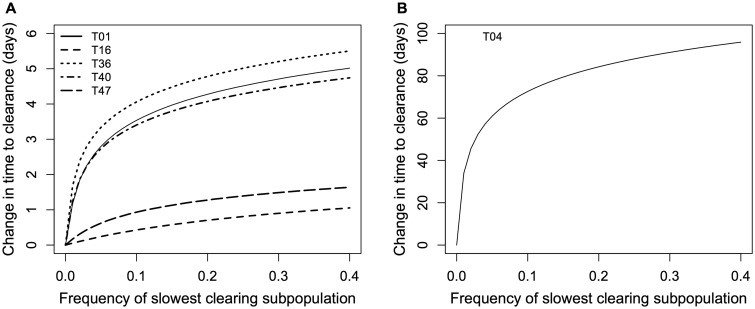
**Predicted effects on clearance time (by PCR) of increasing the initial frequency of the slowest clearing parasite subpopulation in individual infections.** (**A**) The predicted effects on five of the patients from Tanzania in which significant variation in clearance rates was observed. One patient is plotted on its own (**B**) for visibility. (Note that the simulations assume that all relevant rates remain constant, i.e. there are no changes in pharmacokinetics/pharmacodynamics nor other within-host processes, over the relevant timescale. This is likely to be violated over the span of 100 days but does not affect our inference of having detected some very slow clearing subpopulations.)

We simulated infections that capture the relevant clinical details of each of the six Tanzanian patients whose infections showed evidence of variation in clearance rates. We found that modest increases in the initial frequency of the slowest clearing subpopulation observed in each case could lead to large increases in the time to clinical clearance (Fig. 7). All else being equal, these simulations will underestimate the change in clearance time: we compare the predicted clearance time in simulated infections to the clearance time estimated from fitting a linear curve to the total log_e_ parasite density. This latter curve represents a weighted average of the clearance rates of all parasites in an infection, meaning that the slowest clearing subpopulation is exerting its phenotypic effect on that estimate of clearance time. (This is why the predicted difference in time to clearance is 0 h when the frequency of the slowest clearing haplotype is 0.)

**Figure 8.**
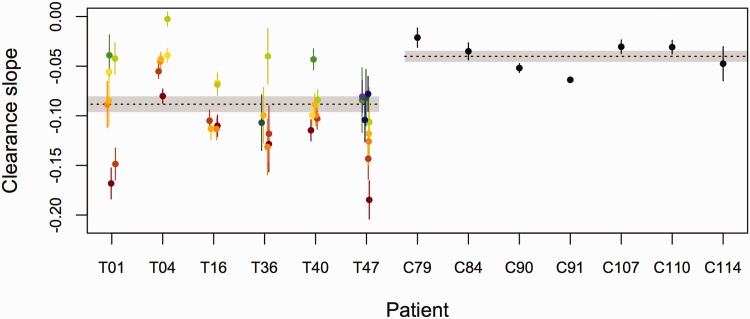
**Comparison of clearance slopes of parasites isolated from patients in Tanzania (patient IDs beginning with T) and Cambodia (patient IDs beginning with C).** For patients from Tanzania, in which significant variation in clearance rates was observed, the predicted clearance slopes ±1 standard error of individual subpopulations are given in different colors. There was no significant variation in infections from Cambodia. The dashed lines (shaded region) represent the mean (±1 standard error of the mean) clearance slopes of patients from each region, as estimated by fitting linear models to the decline in log_e_ total parasite density

Figure 8 compares the parasite clearance slopes estimated from Tanzanian samples with those estimated from Cambodian samples, where resistance is emerging and where these specific patients were responding poorly to treatment by standard measures (microscopy positive at 72 h post-drug treatment; see also Supplementary Fig. S4). Importantly, despite not harboring any of the mutations associated with artemisinin resistance, some of the subpopulations in Tanzania clear as slowly as the parasites in Cambodia, which do harbor those mutations.

## DISCUSSION

Standard parasite clearance curves for malaria infections may underestimate the problem of drug resistance since they are weighted averages of the clearance phenotypes in an individual and therefore struggle to identify resistance when it is rare [7]. By capitalizing on the wealth of quantitative data provided by second generation sequencing, we have developed an approach with the potential to uncover and track resistance in Africa when it is rare by estimating the clearance rates of malaria parasite subpopulations within a given infection. While we found no evidence of variation in clearance rates within infections in Cambodia, where multiplicity of infection is quite low and resistance has already taken hold [2, 4–6], we found evidence of substantial variation in clearance rates in infections in Tanzania. The level of variation we observe is predicted to be important—all else being equal, small changes in the frequency of the slowest clearing parasites could lead to increases in the clearance time of those infections on the order of days. Our data suggest that several parasite subpopulations observed in Tanzania, with no known K13 resistance-associated mutations, clear as slowly as parasites in Cambodia, which are labeled drug resistant and do harbor those mutations. Though quantitative comparisons of clearance rates could be confounded by resistance to the partner drug (which differed across cohorts), differences in starting densities or other factors, clearance slopes in infections in sub-Saharan Africa are likely to underestimate inherent resistance of parasites due to greater host immunity in high transmission settings [39]. The data we report here are consistent with the circulation of rare artemisinin-resistant parasites in Tanzania and therefore urges further study of identified slow clearing subpopulations.

It is possible that processes other than drug resistance could result in slower clearance of some subpopulations within individual infections. Chief among these is differential selection imposed by immune responses. We have previously argued that the power of drug selection would overwhelm these effects [7], especially on timescales as short as one or two replicative cycles, although recent modeling work suggests that immunity exerts a large influence on the clearance of parasites during drug treatment [40]. The relative contribution of immunity and drugs toward generating the variation in clearance rates we observe remains to be determined. This is hard without untreated controls but may be possible with, e.g. a rodent experimental model. For now, it is worth noting that drugs exerted little effect on some of the subpopulations we observed over the timescale studied. For example, the density of the light green subpopulation in T04 changed only very slightly over the course of drug treatment (second row in Fig. 4b). Evading the effects of immunity cannot be the full explanation for the patterns observed for such subpopulations; these parasites must also be evading the effects of drugs. To validate this apparent drug-resistant phenotype, future studies should identify slow clearing parasites and clone them out for *in vitro* assays [9, 21].

Other factors could plausibly affect our estimates of clearance rates. First, lagged effects of drug treatment [20] could have made our linear fits suspect. We do not see any evidence of lag phases in our subpopulation-level data, though these may have been seen with finer sampling intervals (in which case, they could be easily excluded [19]). Second, parasite sequestration or cohort effects could obscure our slope estimates. With sequestration, we would expect parasite densities to drop and then rise in consecutive 24-h periods. We might also expect that the relative abundance of subpopulations would change if there were multiple cohorts of parasites sequestering on different days (i.e. with out-of-phase, 48-h developmental cycles). We find little evidence of these patterns in Tanzania: total parasite densities decline in a fairly linear way and the initially most abundant subpopulation in each infection is always found at all other time points. Some minor subpopulations do disappear and reappear 48 h later (e.g. the tree structures are identical in Fig. 6a and c, but some nodes are missing in b and d). Because those subpopulations become undetectable and we make no assumptions about missing data, our slope estimates are unaffected by the disappearance of minor subpopulations, thus the changes in relative abundance we observe are unlikely to be due to sequestration. (Sequestration might explain the subpopulations that appear only at 72 h post-treatment in Cambodian infections; we do not believe these to be artifacts as their underlying haplotypes differ from the predominant variant in each infection by several base pairs and most match previously described haplotypes. But again, the appearance of these subpopulations does not alter our inferences.) Third, sampling issues leading to underestimating the density of a subpopulation early and/or overestimating its density late in infections would give the impression of slower clearance. Our approach was conservative in a number of ways (see Methods) and while parasite densities at 72 h are low and error in frequency estimates is thus more likely, it is unlikely that the slower clearing subpopulations would be systematically overrepresented only in samples from these later time points. Further, we quantified and accounted for changing error rates in the data.

Since the dilution series control experiments suggest that at low parasite density, amplicon deep sequencing may introduce false haplotypes at low frequency, we used more stringent frequency cutoffs in the low parasitemia samples for our analysis. These haplotypes are unlikely contamination, as the same DNA is represented in all the dilution series samples and the haplotypes did not start to appear until the low parasitemias. There is nothing to suggest that these haplotypes should have a competitive advantage during PCR or sequencing as there are no polymorphisms under the primers, no length differences in the amplicons and no differences in the homoploymer runs between haplotypes. Likely, this represents either mispriming during the PCR or late PCR errors that make it to the sequencer as more PCR product is loaded into the library prep at the low parasitemias. In addition, it is platform independent, as the same phenomenon was observed when the PCR products were sequenced on an Illumina Miseq (data not shown).

All 72-h Tanzanian samples were wild type at all K13 positions, with no evidence of mixed peaks on chromatograph. The subpopulations with a prolonged clearance phenotype seen in Tanzania must therefore have a different mechanism underlying the slow clearance phenotype. In light of recent evidence that the genetic background is important for determining if K13 polymorphisms result in resistance [12, 13], our results support the idea that that resistance is due to multiple genetic loci. Tanzania changed its policy on first-line anti-malarial drugs at the end of 2006 [41]. Our Tanzanian samples were collected before this time [30] and thus, presumably, before the parasites within those samples would have been exposed to significant selective pressure from artemisinin-based therapies. (Interestingly, similar levels of variation in clearance times were recently observed in rodent malaria parasites that had not previously been exposed to artemisinin treatment [42].) Given this apparent standing variation in the absence of drug pressure, we might therefore have predicted a different underlying genetic mechanism for resistance, which is not unheard of in falciparum malaria [14]. Further characterization of the subpopulations with this phenotype in Tanzania could lend important insight into the biology of artemisinin resistance.

The slow clearing subpopulations we find make up a large fraction of the parasites in infections 3 days after drug treatment. Given that patients with such residual parasitemia have previously been shown to have higher transmission potential [43], it is reasonable to predict that the slow clearance we have observed in Tanzania will lead to increased circulation of slow clearing parasites in the host population. Why this has yet to occur, despite continued drug pressure since our samples were collected, may be a combination of increased parasite genetic diversity and competition within infections, the absence of permissive parasite genetic backgrounds [12, 13], low drug coverage and stronger immune responses altering the overall strength of drug selection in sub-Saharan Africa. Our data hint at a key role for within-host competition. The slowest clearance rates we observed in Tanzania were invariably associated with subpopulations that were initially rare in infections (in Fig. 8, the initially rare green and blue haplotypes have the shallowest slopes while the initially abundant red and orange subpopulations have the steepest; see also Supplementary Fig. S5). While this pattern is also consistent with an immune selection model (discussed above), it could indicate that more drug-tolerant strains have a disadvantage within hosts in the absence of treatment. Moreover, population genetic models predict that the effects of any selection imposed by drugs within a host are likely to be obscured by selection acting at the between-host level [44]. If drug-tolerant strains have a competitive disadvantage in the mosquito, then this will also slow the spread of these strains in the host population. For example, tightly linked to the K13 resistance gene is *Pfs47*. This polymorphic gene is responsible for evasion of the mosquito immune system and has been associated with the mosquito species-specific transmission of the parasites [45]. This linkage could limit the spread of resistance mutations between geographic regions where the main vector species differs.

While our approach provides finer resolution than the standard parasite clearance curve on the variation in clearance phenotypes within an infection, each subpopulation-level clearance curve we define still represents a weighted average of that haplotype, which is likely to be comprised of a number of different parasite clones. (Indeed, the slow clearing subpopulations we find in some patients, which are initially rare in those infections, share haplotypes with initially common and relatively fast clearing subpopulations in other patients; see Supplementary Fig. S6). This means that a resistant clone could still be masked in a subpopulation by sensitive parasites that share a haplotype at the sequenced locus. The approach we have outlined will thus not be able to identify every low level and low-frequency resistant parasite that a causative molecular marker would afford. But our approach provides finer resolution than the standard parasite clearance curve on the variation in clearance rates within an infection and is currently the only one that can detect this phenotypic signature of resistance *in vivo* early in individual, polyclonal infections. Further, our data from Tanzania highlights the fact that a complete clinical or molecular definition of resistance may be elusive. The multiplexing approach of using bar-coded primers keeps sequencing costs per sample at a relatively modest level (∼10 dollars a sample for a study of this size) and the rapid data generation on small next-generation sequencers, such as the Ion Torrent or MiSeq, allows for rapid acquisition of data post-amplification. In the end, our approach can aid in refining any working molecular definition of resistance by identifying slow clearing subpopulations for further assessment. Future studies could improve the power of this approach by using multi-locus haplotypes or single cell sequencing, which could provide even finer resolution, and linking this with association studies to locate causal genetic variation. For now, our data show that clearance rates for *Plasmodiun falciparum* parasites in Africa are highly variable, a concerning finding for the emergence of clinically significant resistance to artemisinin.

## Supplementary Material

Supplementary Data
