# Supplementary material for: A deep sequencing tool for partitioning clearance rates following antimalarial treatment in polyclonal infections
Source: Evol Med Public Health. 2016 Jan 27;2016(1):21–36. doi: 10.1093/emph/eov036 (PMC4753362; doi:10.1093/emph/eov036)
Supplement: Supplementary Data [file supp_2016_1_21__index.html]

A deep sequencing tool for partitioning clearance rates following antimalarial treatment in polyclonal infections — Supplementary Data 

# A deep sequencing tool for partitioning clearance rates following antimalarial treatment in polyclonal infections

## Supplementary Data

files

- Supplementary Data - pdf file
