# Supplementary material for: A deep sequencing tool for partitioning clearance rates following antimalarial treatment in polyclonal infections
Source: Evol Med Public Health. 2016 Jan 27;2016(1):21–36. doi: 10.1093/emph/eov036 (PMC4753362; doi:10.1093/emph/eov036)
Supplement: Supplementary Data [file supp_eov036_RevisedSupplementaryMaterial.pdf]

Supplementary Material

To quantify the noise in estimating haplotypes, we generated a sequencing dilution series of control mixtures. Figure S1 shows the frequency of false haplotypes (averaged across replicate runs) for mixtures with different parasite genomes per microliter of sample. We fitted a nonlinear curve to this data and used this curve to predict the frequency below which we expect to see false haplotypes at different input densities. The fitted curve is described by

$$\text{cutoff} = -0.02027 + \frac{0.13282}{\sqrt{\text{density}}}.$$

Table S1 shows the total parasite density, as determined by digital droplet PCR, and total read count for each patient at each time point. For each sample (patient and time point), we used a unique, parasite density-dependent frequency cutoff, requiring a parasite subpopulation (defined by its haplotype at the sequenced locus) to be at an average frequency of at least that cutoff value to be included in subsequent analyses. These cutoffs depended on the parasite density in the sample and are listed in Table S2. At a minimum, we required subpopulations to be at a frequency of 0.005.

The frequencies of all haplotypes for each patient, as determined by sequencing are given in Table S3. These frequencies show the relative abundance of a subpopulation at a given point in time after drug treatment. The sequencing was run in duplicate, so there are two estimates for each subpopulation at each time point. Patients from Tanzania have IDs beginning with T; patients from Cambodia have IDs beginning with C. Subpopulations are ranked by initial abundance.

In the main text, we analyze only data from infections with PCR-detectable parasites at 72 hours post-drug treatment. Here, we present the same analyses for patients from Tanzania with residual parasitemia up to 48 hours post-drug treatment (Figure S2), or with residual parasitemia up to 24 hours post-drug treatment (Figure S3). In total, 14 out of 19 patients from Tanzania demonstrate significant variation in clearance rates across subpopulations. We find the same results when we compare models using AIC instead of anova (Table S4).

As in Figure 8 of the main text, Figure S4 compares the clearance slopes of all subpopulations observed in Tanzanian patients with the slopes observed in Cambodian patients (where there was no variation). Many of the subpopulations in Tanzania are clearing at rates as slow as the Cambodian infections, where the C580Y mutation associated with artemisinin resistance was ubiquitous.

Across the majority of the Tanzanian infections, we find a strong negative correlation between the initial frequency of a subpopulation and its clearance rate (Figure S5), meaning that the slow-clearing subpopulations tend to be rare at the start of treatment.

Figure Legends

Fig. S1. Frequencies of false haplotypes in sequencing dilution series of control mixtures. Also shown is the best-fit nonlinear curve that defined parasite density specific frequency cutoffs which were used to filter the sequencing results from our clinical samples.

Fig. S2. Estimating subpopulation clearance curves from infections in Tanzania, for patients with parasite detectable by PCR up to 48 hours post-drug treatment. (*A*) Relative abundance of parasite subpopulations within patients. For each patient (row) there are two bar graphs representing technical replicates, labeled 1 and 2. Within each patient, individual subpopulations have specific colors and are ranked by initial frequency. (The same color may thus represent different haplotypes in different patients.) (*B*) Densities of individual subpopulations (points) are calculated as the total density by PCR (crosses) multiplied by their relative abundance. Clearance curves are plotted for the total parasite density (dashed lines) and individual subpopulations (colored lines). Statistics reported represent model comparisons; p-values < 0.05 indicate that a more complicated model (one that includes

different slopes for each subpopulation within a patient) explains significantly more variation than a model with a single slope.

Fig. S3. Estimating subpopulation clearance curves from infections in Tanzania, for patients with parasite detectable by PCR up to 24 hours post-drug treatment. (*A*) Relative abundance of parasite subpopulations within patients. (*B*) Densities of individual subpopulations (points) are calculated as the total density by PCR (crosses) multiplied by their relative abundance. Clearance curves are plotted for the total parasite density (dashed lines) and individual subpopulations (colored lines).

Fig. S4. Comparison of clearance slopes of parasites isolated from all patients in Tanzania (patient IDs beginning with T) and Cambodia (patient IDs beginning with C). For patients from Tanzania, in which significant variation in clearance rates was observed, the clearance slopes of individual subpopulations are given in different colors. There was no significant variation in infections from Cambodia. The dashed lines (shaded region) represent the mean (±1 standard error of the mean) clearance slopes across patients from each region, as estimated by fitting linear models to the decline in $\log_e$ total parasite density.

Fig. S5. Relationship between initial subpopulation frequency and clearance slope within patients. Pearson correlation coefficients are reported.

Fig. S6. Overall haplotype frequencies across patients at different time points. Haplotypes are categorized into those that were observed at 72 hours post-treatment (grey bars) and those that were not observed at 72 hours (white bars). Though parasite densities were lowest at 72 hours, and thus sequencing would be more error prone, the haplotypes we found in 72 hour samples occur at other time points as well and are, on average, as common as other haplotypes at those time points. The “population” sample represents the overall frequency of haplotypes across all patients and time points (all samples).

Table S1. Total parasite density, reported as parasite genomes per microliter of extracted DNA, and total read count used for haplotype calling after applying filters, over time for each patient.

Patient	Total parasite density				Total read count			
	0 hrs	24 hrs	48 hrs	72 hrs	0 hrs	24 hrs	48 hrs	72 hrs
T01	24150	57.5	133	15.975	34171	9278	11212	1383
T04	1025	179.5	94.5	14.45	25511	3979	22991	1752
T05	243.5	NA	46.5	NA	23299	NA	24179	NA
T06	600	NA	37.9	NA	27040	NA	11894	NA
T07	38000	NA	93.5	NA	24720	NA	17359	NA
T15	1500	NA	65	NA	29455	NA	12293	NA
T16	27600	1375	133.5	50	32121	20798	28290	2734
T17	6850	214.5	257.5	31.25	45777	8694	14573	1229
T20	16100	NA	41.85	NA	36403	NA	9432	NA
T24	316.5	NA	18.2	NA	37878	NA	10330	NA
T36	24900	107	46.65	8.5	11577	NA	10565	NA
T39	875	232	NA	NA	46341	35164	NA	NA
T40	29300	488.5	250	12.5	20785	19526	13322	1637
T41	1650	49.3	NA	NA	10983	10176	NA	NA
T42	14700	107.5	NA	NA	35154	11712	NA	NA
T43	6750	234.5	NA	NA	18187	14553	NA	NA
T45	21850	NA	45.05	NA	24186	NA	39477	NA
T47	185000	127	27.35	28.75	18243	4379	14074	1259
T48	21550	86	NA	NA	31516	9955	NA	NA
T49	2240	NA	NA	NA	25814	NA	13446	NA
C79	161.5	49.65	80.5	25.15	23974	15699	16726	7528
C84	173.5	67.5	59.5	10.95	4003	3078	1295	967
C90	1445	660	144	37.8	26456	34255	22296	13405
C91	1780	391.5	83	18.15	26214	33019	18990	6710
C107	635	206.5	83.5	75	31756	38638	7382	7176
C110	214.5	168.5	40	29.35	26162	28415	14450	6871
C114	1035	68	35.55	28.65	38121	38281	8180	11495

Table S2. Frequency cutoffs used for individual samples.

Patient	Frequency cutoff			
	0 hrs	24 hrs	48 hrs	72 hrs
**T01**	0.005	0.005	0.005	0.01296
**T04**	0.005	0.005	0.005	0.01467
**T05**	0.005	0.005	0.005	0.005
**T06**	0.005	0.005	0.005	0.005
**T07**	0.005	0.005	0.005	0.005
**T15**	0.005	0.005	0.005	0.005
**T16**	0.005	0.005	0.005	0.005
**T17**	0.005	0.005	0.005	0.005
**T20**	0.005	0.005	0.005	0.005
**T24**	0.005	0.01086	0.005	0.005
**T36**	0.005	0.005	0.005	0.02529
**T39**	0.005	0.005	0.005	0.005
**T40**	0.005	0.005	0.005	0.01730
**T41**	0.005	0.005	0.005	0.005
**T42**	0.005	0.005	0.005	0.005
**T43**	0.005	0.005	0.005	0.005
**T45**	0.005	0.005	0.005	0.005
**T47**	0.005	0.005	0.00513	0.005
**T48**	0.005	0.005	0.005	0.005
**T49**	0.005	0.005	0.005	0.005
**C79**	0.005	0.005	0.005	0.00622
**C84**	0.005	0.005	0.005	0.01987
**C90**	0.005	0.005	0.005	0.005
**C91**	0.005	0.005	0.005	0.01091
**C107**	0.005	0.005	0.005	0.005
**C110**	0.005	0.005	0.005	0.005
**C114**	0.005	0.005	0.005	0.005

Table S3. Frequencies of haplotypes within patients over time.

Patient	Haplotype ID	Run 1 0 hrs	Run 1 24 hrs	Run 1 48 hrs	Run 1 72 hrs	Run 2 0 hrs	Run 2 24 hrs	Run 2 48 hrs	Run 2 72 hrs
T01	1	0.8499	0.9195	0.1079	0.1823	0.8804	0.8840	0.2457	0.1898
T01	2	0.0901	0.0565	0.0855	0.0296	0.0611	0.0719	0.0810	0.0110
T01	3	0.0216	NA	0.1549	NA	0.0162	NA	0.0985	NA
T01	4	0.0151	NA	0.0211	NA	0.0194	NA	0.0167	NA
T01	5	0.0094	NA	0.0961	NA	0.0097	NA	0.0624	NA
T01	6	0.0082	0.0226	0.2190	0.7241	0.0068	0.0178	0.2075	0.7059
T01	7	0.0057	NA	0.0084	NA	0.0063	NA	0.0068	NA
T01	8	NA	0.0015	NA	0.0443	NA	0.0263	NA	0.0025
T01	9	NA	NA	0.1898	0.0197	NA	NA	0.1406	0.0907
T01	10	NA	NA	0.0207	NA	NA	NA	0.0578	NA
T01	11	NA	NA	0.0557	NA	NA	NA	0.0266	NA
T01	12	NA	NA	0.0005	NA	NA	NA	0.0316	NA
T01	13	NA	NA	0.0178	NA	NA	NA	0.0240	NA
T01	14	NA	NA	0.0227	NA	NA	NA	0.0007	NA
T04	1	0.8690	0.9833	0.0585	0.2505	0.8742	0.9835	0.0598	0.4094
T04	2	0.0642	NA	0.0413	0.1495	0.0666	NA	0.0411	0.0555
T04	3	0.0195	NA	0.0234	NA	0.0194	NA	0.0250	NA
T04	4	0.0187	NA	0.0231	NA	0.0179	NA	0.0278	NA
T04	5	0.0109	NA	0.0138	NA	0.0069	NA	0.0150	NA
T04	6	0.0096	0.0167	0.0331	0.5736	0.0083	0.0165	0.0320	0.4618
T04	7	0.0080	NA	NA	NA	0.0068	NA	NA	NA
T04	8	NA	NA	0.7213	NA	NA	NA	0.7027	NA
T04	9	NA	NA	0.0348	NA	NA	NA	0.0369	NA
T04	10	NA	NA	0.0326	NA	NA	NA	0.0293	NA
T04	11	NA	NA	0.0051	NA	NA	NA	0.0124	NA
T04	12	NA	NA	0.0054	0.0088	NA	NA	0.0092	0.0362
T04	13	NA	NA	0.0076	NA	NA	NA	0.0090	NA
T04	14	NA	NA	NA	0.0176	NA	NA	NA	0.0370
T05	1	0.7793	NA	0.1636	NA	0.7950	NA	0.0798	NA
T05	2	0.0506	NA	0.0591	NA	0.0490	NA	0.0113	NA
T05	3	0.0346	NA	0.0287	NA	0.0323	NA	0.0361	NA
T05	4	0.0341	NA	0.0312	NA	0.0312	NA	0.0248	NA
T05	5	0.0298	NA	0.0174	NA	0.0225	NA	0.0063	NA
T05	6	0.0260	NA	0.5679	NA	0.0224	NA	0.3935	NA
T05	7	0.0163	NA	0.0161	NA	0.0157	NA	0.0060	NA
T05	8	0.0125	NA	0.0610	NA	0.0138	NA	0.0150	NA
T05	9	0.0112	NA	0.0181	NA	0.0095	NA	0.0080	NA
T05	10	0.0057	NA	0.0326	NA	0.0087	NA	0.0075	NA
T05	11	NA	NA	0.0043	NA	NA	NA	0.4118	NA
T06	1	0.7896	NA	0.0797	NA	0.7577	NA	0.0752	NA

T06	2	0.0531	NA	0.0115	NA	0.0549	NA	0.0142	NA
T06	3	0.0257	NA	0.0459	NA	0.0462	NA	0.0315	NA
T06	4	0.0295	NA	0.5443	NA	0.0310	NA	0.6509	NA
T06	5	0.0237	NA	0.1965	NA	0.0306	NA	0.0208	NA
T06	6	0.0265	NA	0.0054	NA	0.0228	NA	0.0080	NA
T06	7	0.0186	NA	0.0788	NA	0.0208	NA	0.0834	NA
T06	8	0.0155	NA	0.0195	NA	0.0142	NA	0.0991	NA
T06	9	0.0071	NA	0.0077	NA	0.0118	NA	0.0070	NA
T06	10	0.0108	NA	0.0108	NA	0.0101	NA	0.0099	NA
T07	1	0.5574	NA	NA	NA	0.5616	NA	NA	NA
T07	2	0.1905	NA	0.2143	NA	0.1943	NA	0.4017	NA
T07	3	0.0635	NA	0.0623	NA	0.0593	NA	0.0495	NA
T07	4	0.0582	NA	NA	NA	0.0614	NA	NA	NA
T07	5	0.0432	NA	0.3723	NA	0.0430	NA	0.0058	NA
T07	6	0.0406	NA	0.0090	NA	0.0371	NA	0.0044	NA
T07	7	0.0260	NA	NA	NA	0.0260	NA	NA	NA
T07	8	0.0206	NA	0.3204	NA	0.0173	NA	0.1421	NA
T07	9	NA	NA	0.0046	NA	NA	NA	0.2805	NA
T07	10	NA	NA	0.0008	NA	NA	NA	0.1025	NA
T07	11	NA	NA	0.0163	NA	NA	NA	0.0134	NA
T15	1	0.7944	NA	0.1049	NA	0.8021	NA	0.1880	NA
T15	2	0.0568	NA	0.0751	NA	0.0534	NA	0.0475	NA
T15	3	0.0522	NA	0.0482	NA	0.0535	NA	0.0664	NA
T15	4	0.0282	NA	0.0602	NA	0.0282	NA	0.0076	NA
T15	5	0.0204	NA	0.0457	NA	0.0141	NA	0.0140	NA
T15	6	0.0141	NA	NA	NA	0.0194	NA	NA	NA
T15	7	0.0150	NA	0.1995	NA	0.0127	NA	0.2030	NA
T15	8	0.0105	NA	0.0617	NA	0.0089	NA	0.0094	NA
T15	9	0.0084	NA	0.0692	NA	0.0078	NA	0.0949	NA
T15	10	NA	NA	0.2722	NA	NA	NA	0.2938	NA
T15	11	NA	NA	0.0250	NA	NA	NA	0.0504	NA
T15	12	NA	NA	0.0250	NA	NA	NA	0.0003	NA
T15	13	NA	NA	0.0133	NA	NA	NA	0.0247	NA
T16	1	0.6990	0.7700	0.6756	0.1343	0.7309	0.7822	0.6902	0.1336
T16	2	0.2527	0.2159	0.2348	0.0567	0.2177	0.2066	0.2587	0.0646
T16	3	0.0227	NA	0.0217	NA	0.0247	NA	0.0210	NA
T16	4	0.0105	NA	0.0141	NA	0.0145	NA	0.0089	NA
T16	5	0.0081	NA	0.0428	0.1563	0.0065	NA	0.0088	0.0124
T16	6	0.0070	NA	0.0062	0.0294	0.0056	NA	0.0063	0.0578
T16	7	NA	0.0141	NA	0.6149	NA	0.0112	NA	0.6940
T16	8	NA	NA	0.0049	NA	NA	NA	0.0061	NA
T16	9	NA	NA	NA	0.0031	NA	NA	NA	0.0202
T16	10	NA	NA	NA	0.0052	NA	NA	NA	0.0174

T17	1	0.6691	0.6311	0.1179	0.1359	0.2939	0.5707	0.2936	0.2932
T17	2	0.0206	NA	0.0579	NA	0.2000	NA	0.0921	NA
T17	3	0.1432	NA	0.0369	NA	0.0122	NA	0.0059	NA
T17	4	0.0313	0.0126	0.4139	0.6641	0.1102	0.0124	0.0702	0.4267
T17	5	0.0439	NA	0.0582	0.1462	0.1061	NA	0.2935	0.0787
T17	6	0.0293	NA	0.0408	NA	0.0898	NA	0.0004	NA
T17	7	0.0093	NA	0.0435	NA	0.0776	NA	0.0347	NA
T17	8	0.0113	NA	0.0150	NA	0.0367	NA	0.0136	NA
T17	9	0.0293	NA	0.0324	NA	0.0367	NA	0.0254	NA
T17	10	0.0048	NA	0.0052	NA	0.0204	NA	0.0070	NA
T17	11	0.0079	NA	0.0109	NA	0.0163	NA	0.0081	NA
T17	12	NA	0.3466	0.0506	0.0256	NA	0.3971	0.0560	0.0405
T17	13	NA	0.0096	NA	NA	NA	0.0197	NA	NA
T17	14	NA	NA	0.0476	0.0077	NA	NA	0.0576	0.0310
T17	15	NA	NA	0.0271	NA	NA	NA	0.0408	NA
T17	16	NA	NA	0.0226	NA	NA	NA	0.0006	NA
T17	17	NA	NA	0.0193	NA	NA	NA	0.0006	NA
T17	18	NA	NA	NA	0.0205	NA	NA	NA	0.1299
T20	1	0.6617	NA	0.2766	NA	0.6511	NA	0.5107	NA
T20	2	0.2066	NA	NA	NA	0.2022	NA	NA	NA
T20	3	0.0907	NA	0.0121	NA	0.0932	NA	0.0102	NA
T20	4	0.0259	NA	0.2059	NA	0.0412	NA	0.0476	NA
T20	5	0.0085	NA	0.0141	NA	0.0056	NA	0.0173	NA
T20	6	0.0066	NA	0.0091	NA	0.0066	NA	0.0080	NA
T20	7	NA	NA	0.1947	NA	NA	NA	0.3731	NA
T20	8	NA	NA	0.1645	NA	NA	NA	0.0011	NA
T20	9	NA	NA	0.0666	NA	NA	NA	0.0094	NA
T20	10	NA	NA	0.0405	NA	NA	NA	0.0039	NA
T20	11	NA	NA	0.0091	NA	NA	NA	0.0107	NA
T20	12	NA	NA	0.0069	NA	NA	NA	0.0080	NA
T24	1	0.0117	NA	0.1111	NA	0.3073	NA	0.1203	NA
T24	2	0.2825	NA	NA	NA	0.1188	NA	NA	NA
T24	3	0.2724	NA	NA	NA	0.1154	NA	NA	NA
T24	4	0.0972	NA	NA	NA	0.0840	NA	NA	NA
T24	5	0.0103	NA	0.6440	NA	0.0846	NA	0.6518	NA
T24	6	0.0656	NA	0.0489	NA	0.0215	NA	0.0398	NA
T24	7	0.0597	NA	NA	NA	0.0322	NA	NA	NA
T24	8	0.0470	NA	NA	NA	0.0577	NA	NA	NA
T24	9	0.0037	NA	NA	NA	0.0393	NA	NA	NA
T24	10	0.0369	NA	NA	NA	0.0308	NA	NA	NA
T24	11	0.0195	NA	NA	NA	0.0347	NA	NA	NA
T24	12	0.0336	NA	0.0145	NA	0.0184	NA	0.0156	NA
T24	13	0.0241	NA	NA	NA	0.0106	NA	NA	NA

T24	14	0.0234	NA	NA	NA	0.0239	NA	NA	NA
T24	15	0.0076	NA	0.0648	NA	0.0130	NA	0.0672	NA
T24	16	0.0047	NA	NA	NA	0.0079	NA	NA	NA
T24	17	NA	NA	0.0554	NA	NA	NA	0.0420	NA
T24	18	NA	NA	0.0345	NA	NA	NA	0.0306	NA
T24	19	NA	NA	0.0269	NA	NA	NA	0.0326	NA
T36	1	0.8773	0.9757	0.0553	0.2349	0.8610	0.9767	0.2470	0.2336
T36	2	0.0543	NA	0.1900	NA	0.0613	NA	0.0597	NA
T36	3	0.0208	NA	0.0299	NA	0.0249	NA	0.0163	NA
T36	4	0.0132	NA	0.5250	NA	0.0203	NA	0.0104	NA
T36	5	0.0110	NA	NA	NA	0.0123	NA	NA	NA
T36	6	0.0089	0.0243	0.0675	0.7560	0.0082	0.0233	0.4055	0.6524
T36	7	0.0079	NA	NA	NA	0.0057	NA	NA	NA
T36	8	0.0068	NA	0.0557	NA	0.0062	NA	0.0075	NA
T36	9	NA	NA	0.0285	0.0092	NA	NA	0.1243	0.1140
T36	10	NA	NA	0.0338	NA	NA	NA	0.0897	NA
T36	11	NA	NA	0.0028	NA	NA	NA	0.0390	NA
T36	12	NA	NA	0.0114	NA	NA	NA	0.0007	NA
T39	1	0.4969	0.8127	NA	NA	0.5262	0.8914	NA	NA
T39	2	0.2989	0.1567	NA	NA	0.3011	0.0832	NA	NA
T39	3	0.1377	NA	NA	NA	0.1159	NA	NA	NA
T39	4	0.0195	NA	NA	NA	0.0096	NA	NA	NA
T39	5	0.0164	NA	NA	NA	0.0126	NA	NA	NA
T39	6	0.0057	NA	NA	NA	0.0093	NA	NA	NA
T39	7	0.0055	NA	NA	NA	0.0075	NA	NA	NA
T39	8	0.0074	NA	NA	NA	0.0070	NA	NA	NA
T39	9	0.0071	NA	NA	NA	0.0045	NA	NA	NA
T39	10	0.0050	NA	NA	NA	0.0064	NA	NA	NA
T39	11	NA	0.0306	NA	NA	NA	0.0255	NA	NA
T40	1	0.8954	0.9461	0.8609	0.2432	0.8845	0.9279	0.7846	0.3309
T40	2	0.0320	0.0378	0.0078	0.0396	0.0358	0.0571	0.0810	0.0268
T40	3	0.0276	NA	0.0273	NA	0.0214	NA	0.0214	NA
T40	4	0.0155	NA	0.0193	NA	0.0192	NA	0.0423	NA
T40	5	0.0094	NA	0.0092	NA	0.0166	NA	0.0170	NA
T40	6	0.0131	NA	0.0162	0.1550	0.0141	NA	0.0315	0.0407
T40	7	0.0071	0.0162	0.0594	0.5532	0.0085	0.0150	0.0222	0.5287
T40	8	NA	NA	NA	0.0090	NA	NA	NA	0.0730
T41	1	0.4695	0.7022	NA	NA	0.4922	0.6963	NA	NA
T41	2	0.2714	0.0588	NA	NA	0.2497	0.1001	NA	NA
T41	3	0.1554	NA	NA	NA	0.1543	NA	NA	NA
T41	4	0.0356	NA	NA	NA	0.0411	NA	NA	NA
T41	5	0.0179	0.0059	NA	NA	0.0191	0.0132	NA	NA
T41	6	0.0185	0.0695	NA	NA	0.0133	0.0609	NA	NA

T41	7	0.0165	0.0055	NA	NA	0.0180	0.0070	NA	NA
T41	8	0.0077	NA	NA	NA	0.0065	NA	NA	NA
T41	9	0.0074	0.0449	NA	NA	0.0060	0.0376	NA	NA
T41	10	NA	0.0478	NA	NA	NA	0.0434	NA	NA
T41	11	NA	0.0336	NA	NA	NA	0.0159	NA	NA
T41	12	NA	0.0319	NA	NA	NA	0.0256	NA	NA
T42	1	0.5369	0.4637	NA	NA	0.6829	0.3889	NA	NA
T42	2	0.4631	0.3511	NA	NA	0.3171	0.3731	NA	NA
T42	3	NA	0.0077	NA	NA	NA	0.0513	NA	NA
T42	4	NA	0.0471	NA	NA	NA	0.0367	NA	NA
T42	5	NA	0.0431	NA	NA	NA	0.0231	NA	NA
T42	6	NA	0.0144	NA	NA	NA	0.0338	NA	NA
T42	7	NA	0.0223	NA	NA	NA	0.0094	NA	NA
T42	8	NA	0.0153	NA	NA	NA	0.0220	NA	NA
T42	9	NA	0.0185	NA	NA	NA	0.0134	NA	NA
T42	10	NA	0.0073	NA	NA	NA	0.0184	NA	NA
T42	11	NA	0.0054	NA	NA	NA	0.0160	NA	NA
T42	12	NA	0.0041	NA	NA	NA	0.0138	NA	NA
T43	1	0.7441	0.3327	NA	NA	0.7242	0.4404	NA	NA
T43	2	0.1724	0.5396	NA	NA	0.1789	0.4530	NA	NA
T43	3	0.0543	0.0723	NA	NA	0.0661	0.0643	NA	NA
T43	4	0.0189	0.0323	NA	NA	0.0224	0.0150	NA	NA
T43	5	0.0104	0.0136	NA	NA	0.0084	0.0140	NA	NA
T43	6	NA	0.0095	NA	NA	NA	0.0134	NA	NA
T45	1	0.5721	NA	0.0483	NA	0.5926	NA	0.1353	NA
T45	2	0.2568	NA	NA	NA	0.2856	NA	NA	NA
T45	3	0.0314	NA	0.1274	NA	0.0259	NA	0.0662	NA
T45	4	0.0271	NA	0.0312	NA	0.0219	NA	0.0270	NA
T45	5	0.0198	NA	0.1557	NA	0.0112	NA	0.1725	NA
T45	6	0.0194	NA	0.1884	NA	0.0098	NA	0.2152	NA
T45	7	0.0164	NA	0.0645	NA	0.0127	NA	0.0038	NA
T45	8	0.0160	NA	0.1462	NA	0.0084	NA	0.0617	NA
T45	9	0.0146	NA	NA	NA	0.0100	NA	NA	NA
T45	10	0.0103	NA	0.0955	NA	0.0060	NA	0.0662	NA
T45	11	0.0099	NA	0.0132	NA	0.0100	NA	0.0150	NA
T45	12	0.0061	NA	0.0325	NA	0.0059	NA	0.0072	NA
T45	13	NA	NA	0.0820	NA	NA	NA	0.1639	NA
T45	14	NA	NA	0.0112	NA	NA	NA	0.0403	NA
T45	15	NA	NA	0.0039	NA	NA	NA	0.0255	NA
T47	1	0.6423	0.5232	0.0856	0.0961	0.6512	0.4962	0.4843	0.1740
T47	2	0.1332	0.3133	0.0203	0.0366	0.1307	0.3119	0.0095	0.1131
T47	3	0.0765	0.0946	0.0633	0.0183	0.0712	0.1181	0.0625	0.0365
T47	4	0.0365	NA	0.0248	NA	0.0345	NA	0.0263	NA

T47	5	0.0246	NA	0.0128	NA	0.0260	NA	0.0505	NA
T47	6	0.0174	0.0261	0.0281	NA	0.0195	0.0326	0.0037	NA
T47	7	0.0172	NA	0.0150	NA	0.0170	NA	0.0158	NA
T47	8	0.0160	NA	0.0099	NA	0.0118	NA	0.0089	NA
T47	9	0.0110	NA	0.0701	0.1716	0.0117	NA	0.0676	0.0097
T47	10	0.0108	0.0429	0.1367	0.6522	0.0096	0.0411	0.1255	0.5255
T47	11	0.0089	NA	0.3962	0.0046	0.0105	NA	0.1127	0.0487
T47	12	0.0057	NA	NA	NA	0.0062	NA	NA	NA
T47	13	NA	NA	0.0978	NA	NA	NA	0.0058	NA
T47	14	NA	NA	0.0004	NA	NA	NA	0.0251	NA
T47	15	NA	NA	0.0208	0.0206	NA	NA	0.0012	0.0925
T47	16	NA	NA	0.0183	NA	NA	NA	0.0007	NA
T48	1	0.6991	0.4735	NA	NA	0.6778	0.4913	NA	NA
T48	2	0.2133	0.1544	NA	NA	0.2614	0.1922	NA	NA
T48	3	0.0529	0.0477	NA	NA	0.0334	0.0402	NA	NA
T48	4	0.0346	0.0305	NA	NA	0.0274	0.0294	NA	NA
T48	5	NA	0.0535	NA	NA	NA	0.0786	NA	NA
T48	6	NA	0.0665	NA	NA	NA	0.0335	NA	NA
T48	7	NA	0.0530	NA	NA	NA	0.0107	NA	NA
T48	8	NA	0.0164	NA	NA	NA	0.0382	NA	NA
T48	9	NA	0.0327	NA	NA	NA	0.0316	NA	NA
T48	10	NA	0.0151	NA	NA	NA	0.0257	NA	NA
T48	11	NA	0.0182	NA	NA	NA	0.0209	NA	NA
T48	12	NA	0.0141	NA	NA	NA	0.0004	NA	NA
T48	13	NA	0.0126	NA	NA	NA	0.0068	NA	NA
T48	14	NA	0.0118	NA	NA	NA	0.0004	NA	NA
C79	1	1.0000	0.9930	1.0000	0.9018	1.0000	0.9920	1.0000	0.9481
C79	2	NA	0.0070	NA	0.0460	NA	0.0080	NA	0.0270
C79	3	NA	NA	NA	0.0384	NA	NA	NA	0.0013
C79	4	NA	NA	NA	0.0131	NA	NA	NA	0.0117
C79	5	NA	NA	NA	0.0008	NA	NA	NA	0.0119
C84	1	1.0000	1.0000	1.0000	0.9405	1.0000	1.0000	1.0000	0.8814
C84	2	NA	NA	NA	0.0238	NA	NA	NA	0.0475
C84	3	NA	NA	NA	0.0149	NA	NA	NA	0.0373
C84	4	NA	NA	NA	0.0208	NA	NA	NA	0.0339
C90	1	1.0000	1.0000	1.0000	0.9853	1.0000	1.0000	1.0000	0.9713
C90	2	NA	NA	NA	0.0013	NA	NA	NA	0.0184
C90	3	NA	NA	NA	0.0134	NA	NA	NA	0.0103
C91	1	1.0000	1.0000	1.0000	0.9824	1.0000	1.0000	1.0000	0.9899
C91	2	NA	NA	NA	0.0176	NA	NA	NA	0.0101
C107	1	1.0000	1.0000	1.0000	0.8942	1.0000	1.0000	1.0000	0.9335
C107	2	NA	NA	NA	0.1058	NA	NA	NA	0.0665
C110	1	1.0000	1.0000	1.0000	0.9176	1.0000	1.0000	1.0000	0.7746

C110	2	NA	NA	NA	0.0039	NA	NA	NA	0.1644
C110	3	NA	NA	NA	0.0532	NA	NA	NA	0.0303
C110	4	NA	NA	NA	0.0254	NA	NA	NA	0.0308
C114	1	0.9929	0.9914	1.0000	0.8307	0.9917	0.9904	1.0000	0.7125
C114	2	0.0071	0.0086	NA	0.0303	0.0083	0.0096	NA	0.2090
C114	3	NA	NA	NA	0.0759	NA	NA	NA	0.0641
C114	4	NA	NA	NA	0.0435	NA	NA	NA	0.0102
C114	5	NA	NA	NA	0.0195	NA	NA	NA	0.0041

Table S4. Comparison of model selection approaches. AIC scores for fitting a model with different slopes and intercepts for different parasite subpopulations to data from individual patients (Model 1), and for a simpler model that includes different intercepts but a single slope (Model 2).

Last detected parasites by PCR	Patient ID	AIC Model 1	AIC Model 2	Model selection by AIC*	Model selection by anova†
72 hours post-treatment	T01	131.2	164.6	1	1
	T04	66.2	101.6	1	1
	T16	96.7	106.9	1	1
	T17	198	194.3	2	2
	T36	120.8	129.3	1	1
	T40	114.1	130.5	1	1
	T47	225.8	249.5	1	1
48 hours post-treatment	T05	55.5	110.9	1	1
	T06	67.2	120.7	1	1
	T07	63.8	69.3	2	2
	T15	65.9	94.5	1	1
	T20	26.9	52.9	1	1
	T24	61.7	67.2	2	2
	T45	86.9	119.9	1	1
24 hours post-treatment	T39	3.5	16.1	1	1
	T41	5.7	60.8	1	1
	T42	-1.0	-0.5	2	2
	T43	1.1	23.6	1	1
	T48	-7.2	-5.5	2	2

* The model with the lowest AIC value provides a better fit. We use the criteria that a more complex model is selected only if it has an Akaike weight greater than 0.95 (interpreted as a 95% chance that Model 1 is the best model for the data[1]), which occurs when $AIC_{Model2} – AIC_{Model1} > 5.9$.
† This method compares the residual deviation left in the two models. Selecting the more complicated model (Model 1) is only justified when significantly more variation is explained. Results are as reported in the text and figures.
[1] Johnson JB, Omland KS. Model selection in ecology and evolution. *Trends Ecol Evol* 2004**;19**:101-108.

Figure S1

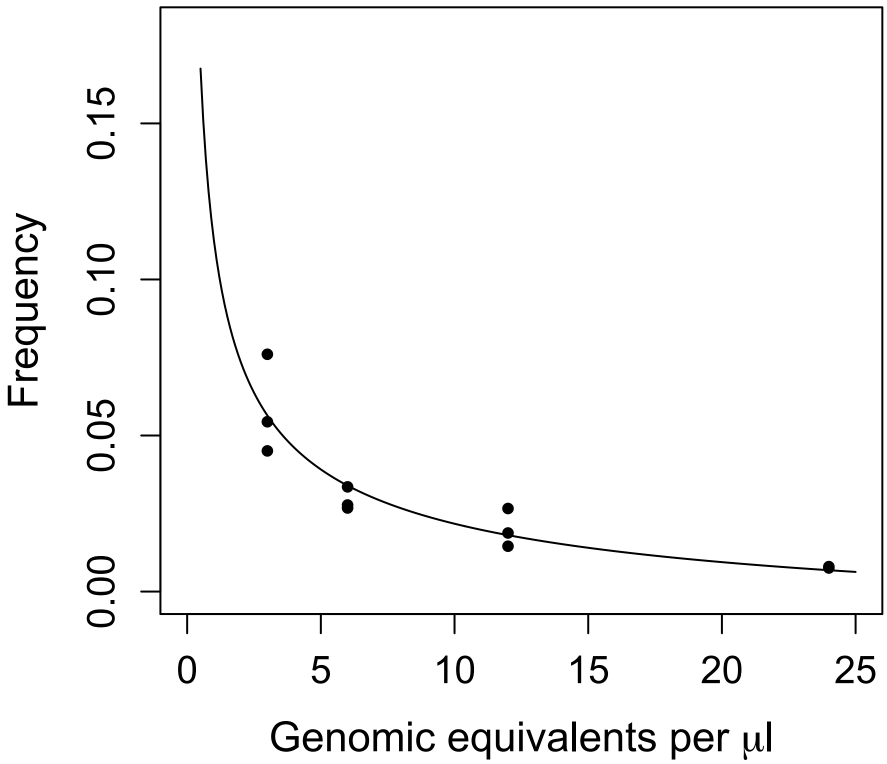

Frequency
0.00
0.05
0.10
0.15
0
5
10
15
20
25
Genomic equivalents per μl

Figure S2

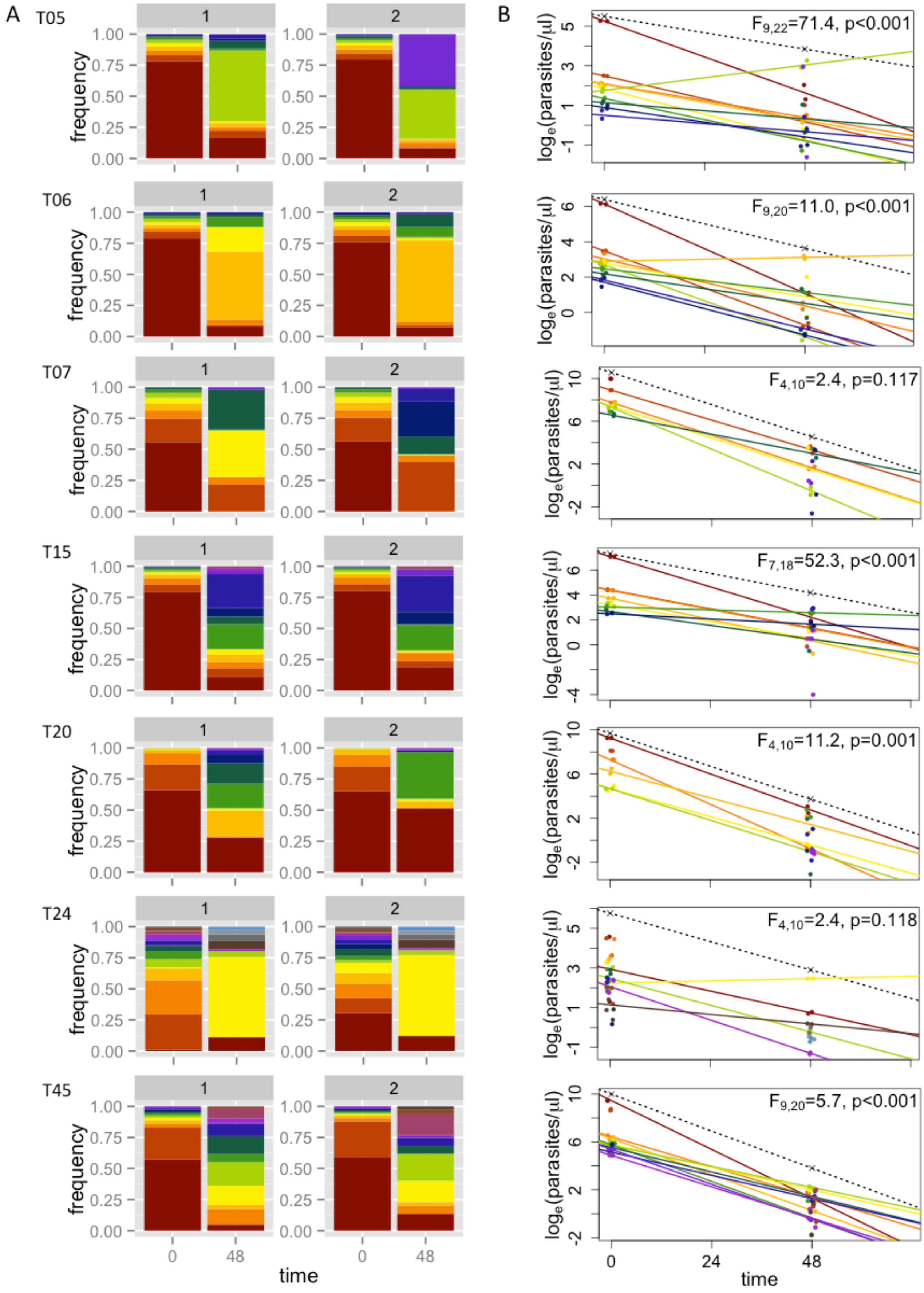
A
B
T05
T06
T07
T15
T20
T24
T45
1
2
frequency
0.00
0.25
0.50
0.75
1.00
0
48
time
loge(parasites/μl)
0
24
48
time
F9,22=71.4, p<0.001
F9,20=11.0, p<0.001
F4,10=2.4, p=0.117
F7,18=52.3, p<0.001
F4,10=11.2, p=0.001
F4,10=2.4, p=0.118
F9,20=5.7, p<0.001

Figure S3

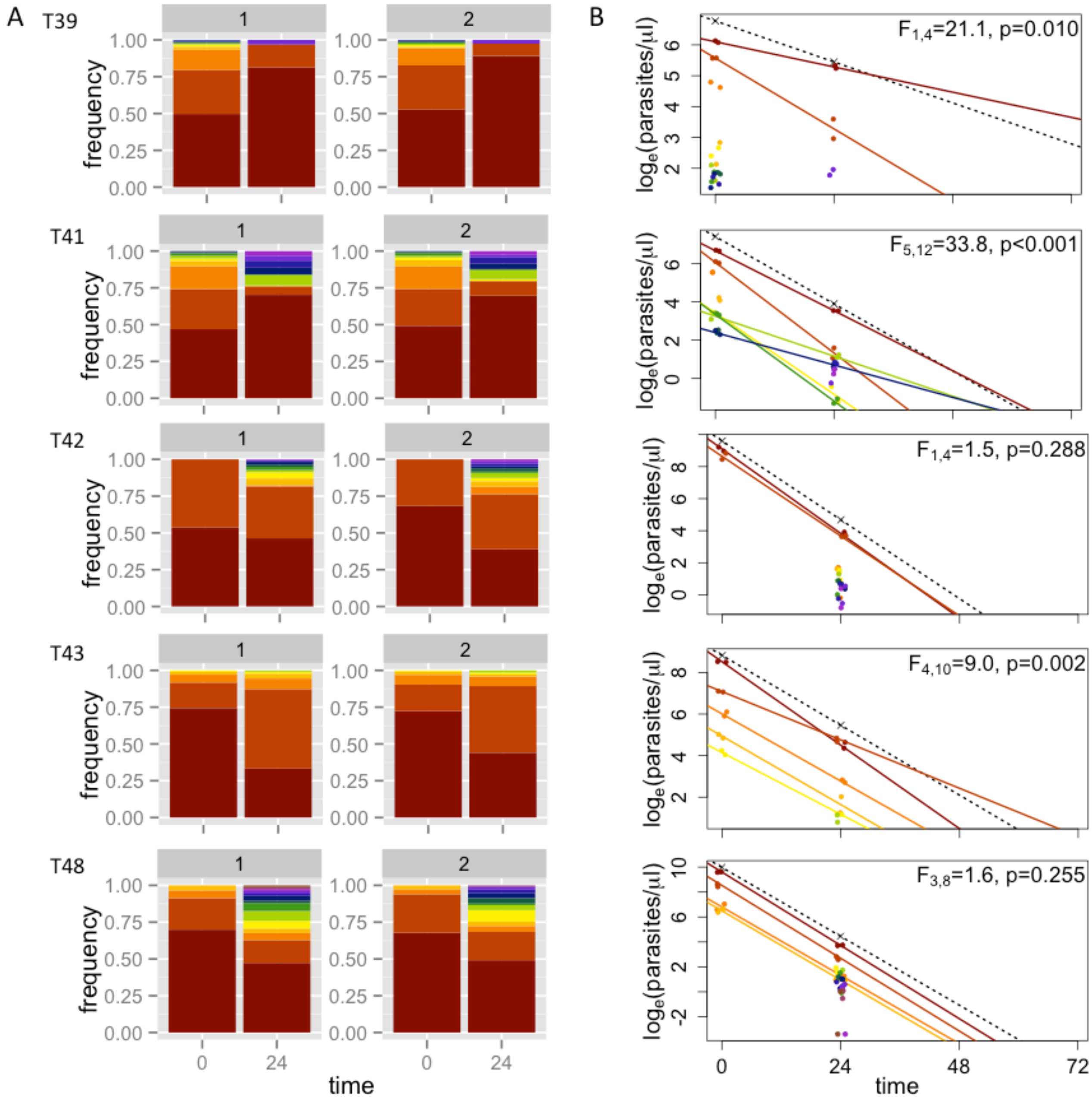
A
T39
T41
T42
T43
T48
1
2
frequency
1.00
0.75
0.50
0.25
0.00
0
24
time
B
log$_e$(parasites/μl)
$F_{1,4}$=21.1, p=0.010
$F_{5,12}$=33.8, p<0.001
$F_{1,4}$=1.5, p=0.288
$F_{4,10}$=9.0, p=0.002
$F_{3,8}$=1.6, p=0.255
0
24
48
72
time

Figure S4

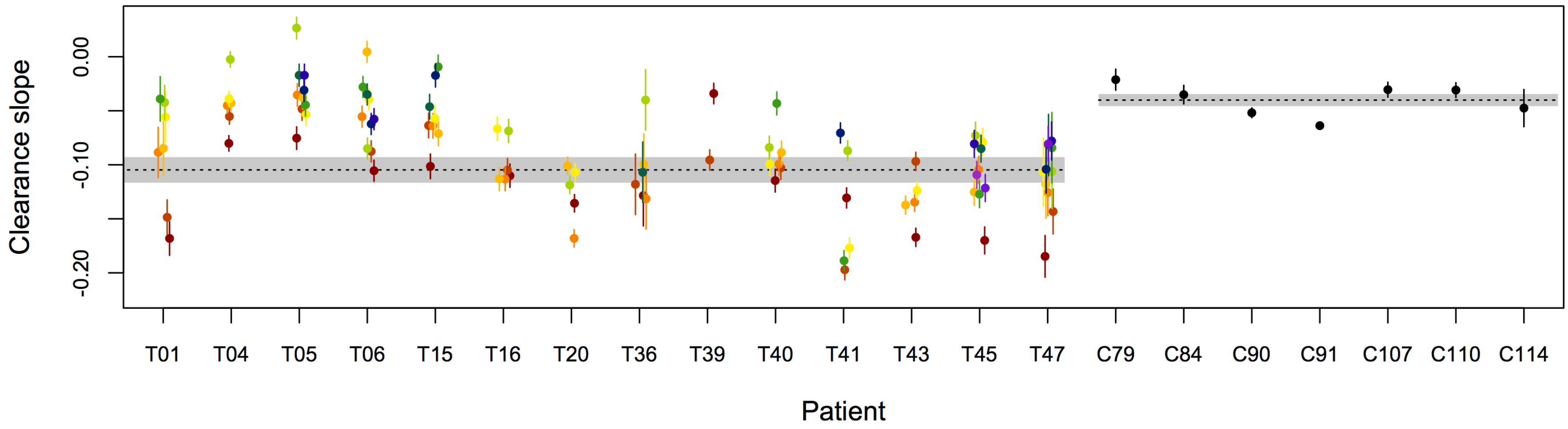

Clearance slope
0.00
-0.10
-0.20
T01
T04
T05
T06
T15
T16
T20
T36
T39
T40
T41
T43
T45
T47
C79
C84
C90
C91
C107
C110
C114
Patient

Figure S5

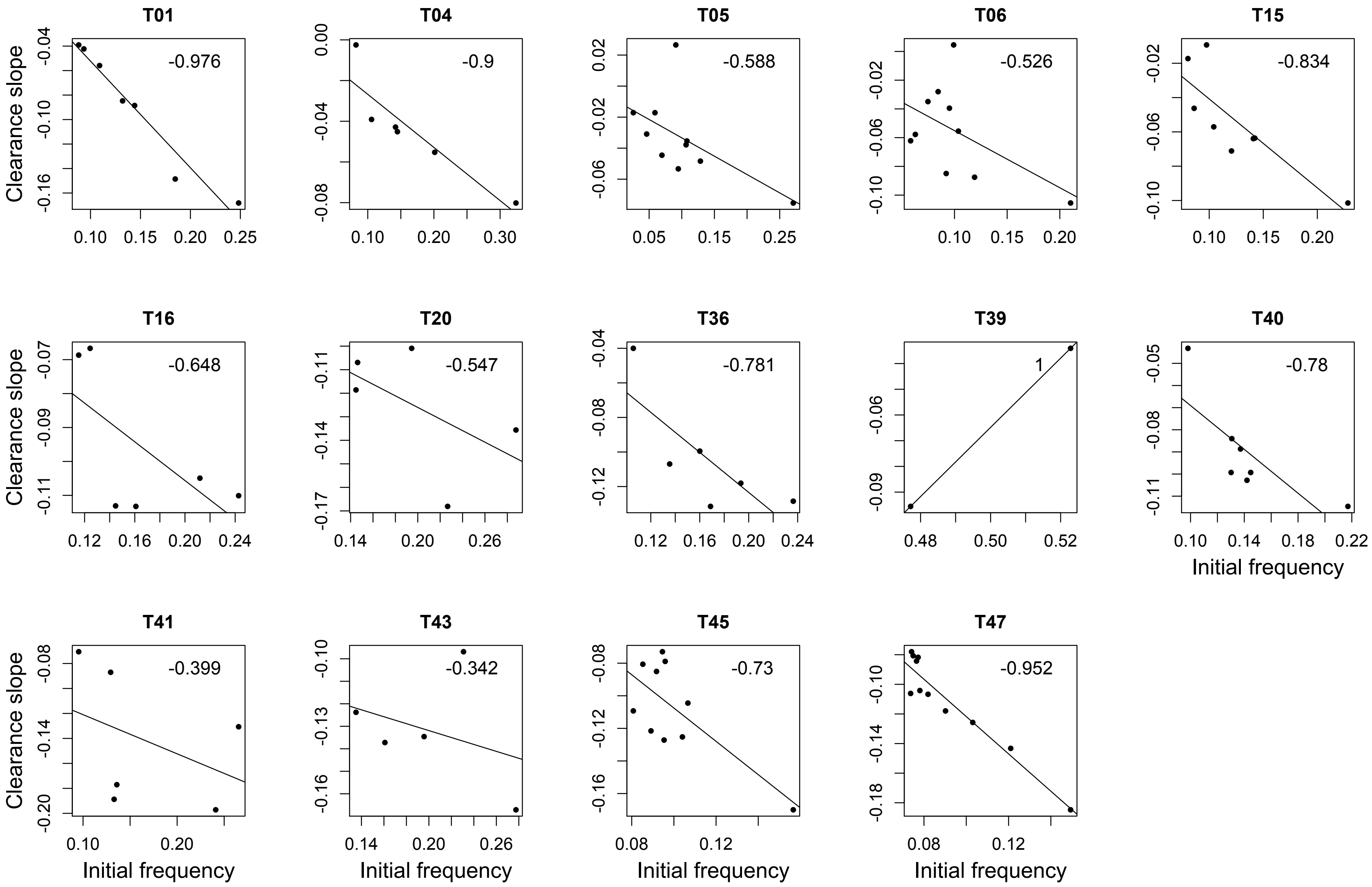

T01
-0.976
T04
-0.9
T05
-0.588
T06
-0.526
T15
-0.834
T16
-0.648
T20
-0.547
T36
-0.781
T39
1
T40
-0.78
T41
-0.399
T43
-0.342
T45
-0.73
T47
-0.952
Clearance slope
Initial frequency

Figure S6

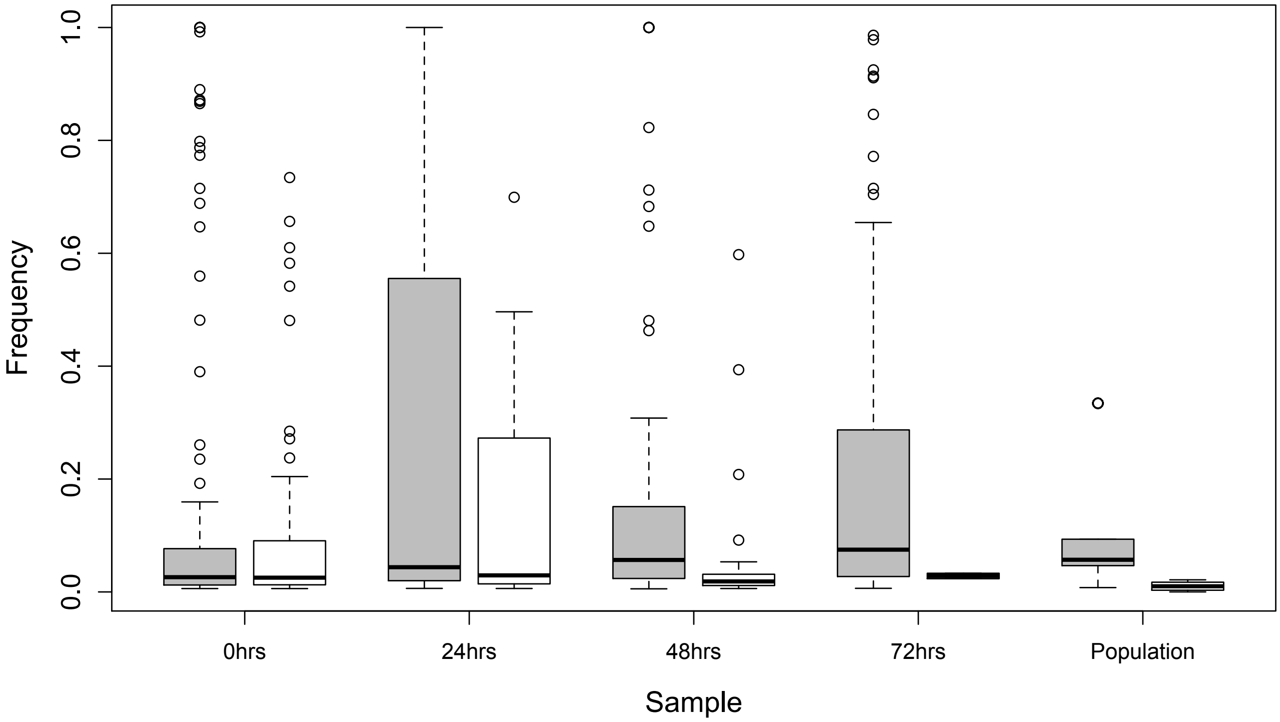

0.0
0.2
0.4
0.6
0.8
1.0
Frequency
0hrs
24hrs
48hrs
72hrs
Population
Sample
